# Dietary fat quality impacts genome-wide DNA methylation patterns in a cross-sectional study of Greek preadolescents

**DOI:** 10.1038/ejhg.2014.139

**Published:** 2014-07-30

**Authors:** Sarah Voisin, Markus S Almén, George Moschonis, George P Chrousos, Yannis Manios, Helgi B Schiöth

**Affiliations:** 1 Department of Neuroscience, Functional Pharmacology, Uppsala University, Uppsala, Sweden; 2 Department of Nutrition and Dietetics, Harokopio University, Athens, Greece; 3 First Department of Pediatrics, Athens University Medical School, Aghia Sophia Children's Hospital, Athens, Greece

## Abstract

The type and the amount of dietary fat have a significant influence on the metabolic pathways involved in the development of obesity, metabolic syndrome, diabetes type 2 and cardiovascular diseases. However, it is unknown to what extent this modulation is achieved through DNA methylation. We assessed the effects of cholesterol intake, the proportion of energy intake derived from fat, the ratio of polyunsaturated fatty acids (PUFA) to saturated fatty acids (SFA), the ratio of monounsaturated fatty acids (MUFA) to SFA, and the ratio of MUFA+PUFA to SFA on genome-wide DNA methylation patterns in normal-weight and obese children. We determined the genome-wide methylation profile in the blood of 69 Greek preadolescents (∼10 years old) as well as their dietary intake for two consecutive weekdays and one weekend day. The methylation levels of one CpG island shore and four sites were significantly correlated with total fat intake. The methylation levels of 2 islands, 11 island shores and 16 sites were significantly correlated with PUFA/SFA; of 9 islands, 26 island shores and 158 sites with MUFA/SFA; and of 10 islands, 40 island shores and 130 sites with (MUFA+PUFA)/SFA. We found significant gene enrichment in 34 pathways for PUFA/SFA, including the leptin pathway, and a significant enrichment in 5 pathways for (MUFA+PUFA)/SFA. Our results suggest that specific changes in DNA methylation may have an important role in the mechanisms involved in the physiological responses to different types of dietary fat.

## Introduction

According to the World Health Organization^1^, worldwide obesity has nearly doubled since 1980, resulting in an increase in cardiovascular diseases and diabetes type 2. One of the possible causes to this negative development is the increase of consumption of energy-dense foods that are high in fat. Dietary guidelines do not only recommend to eat a moderate amount of fat, but they also recommend to consume the right type of fat.^2^ Fatty acids include saturated fatty acids (SFA), monounsaturated fatty acids (MUFA) or polyunsaturated fatty acids (PUFA), and their structural differences explain why they have different biological effects.^3^ Consuming PUFA or MUFA instead of SFA is known to improve the blood lipid profile.^4^ Moreover, consumption of SFA in place of MUFA may worsen glucose-insulin homeostasis.^5^ Finally, replacing SFA with PUFA has been reported to lower coronary heart disease risk.^6^

Some of the effects of the qualitative and quantitative aspects of fat intake have been imputed to a modification of the transcription of key genes involved in pathways related to lipid and glucose metabolism, and/or inflammation.^7^ The regulation of gene expression can be achieved by mechanisms other than changes in the nucleotide sequence, namely epigenetic processes. Such processes are responsible for the establishment, maintenance, and reversal of metastable transcriptional states.^8^ One major example of such processes is the methylation of cytosine, usually at CpG dinucleotides, called DNA methylation. Regions rich in CpGs are called ‘CpG islands' and are mostly unmethylated when located in the promoter of active genes. Conversely, methylated promoters are associated with reduced gene expression.^9^

Five studies have investigated the link between DNA methylation and fat intake in humans, but the methylation assays in those studies were limited to only few key genes. One study found a significantly higher methylation in the peroxisome proliferator-activated receptor coactivator-1 gene (*PPARGC1A*) in high-fat overfed men.^10^ Another study found that the clock circadian regulator gene (*CLOCK*) methylation was negatively associated with MUFA intake, but positively associated with PUFA intake.^11^ A third study showed that higher n-6 PUFA intake was associated with lower methylation in the promoter of tumor necrosis factor-*α* (*TNFα*).^12^ A fourth study found no significant correlation between a diet rich in fat and sucrose, and methylation of hydroxyacyl-coenzyme A dehydrogenase (*HADH*) and glucokinase (*GCK*) genes.^13^ The fifth paper reported a lack of correlation between four diets enriched in different types of fat and the methylation levels of leptin (*LEP*), leptin receptor (*LEPR*), and pro-opiomelanocortin (*POMC*) genes.^14^

Here we explore the genome-wide DNA methylation profiles of Greek preadolescents with respect to parameters related to dietary fat quantity, and dietary fat quality. To our knowledge, this is the first time that parameters related to both quantitative and qualitative aspects of fat intake with respect to DNA methylation are investigated at a genome-wide scale. Moreover, no such studies have been performed in children.

## Materials and Methods

Genome-wide changes of DNA methylation pattern associated with parameters related to fat intake were assessed. Two variables related to dietary fat quantity (proportion of energy intake derived from fat, cholesterol intake) and three related to dietary fat quality (MUFA/SFA, PUFA/SFA and (MUFA+PUFA)/SFA) were analyzed. A linear model that explains the methylation level for each CpG site/island corrected for gender, weight category, Tanner stage (an estimation of physical development), and white blood cell count was utilized. The ratios between the unsaturated and saturated fatty acid intakes were preferred to their individual values, as they have been reported to have antagonistic effects. A higher fatty acids ratio would account for a ‘healthier' fatty acid intake profile, while a lower ratio would account for an ‘unhealthier' fatty acid intake profile.

### Ethics

All participants and their guardians gave informed written consent and the study was approved by the Greek Ministry of National Education (7055/C7-Athens, 19-01-2007) and the Ethical Committee of Harokopio University (16/ Athens, 19-12-2006).

### Subjects

The ‘Healthy Growth Study' was a cross-sectional epidemiological study initiated in May 2007. Approval to conduct the study was granted by the Greek Ministry of National Education (7055/C7-Athens, 19-01-2007) and the Ethics Committee of Harokopio University of Athens (16/Athens, 19-12-2006). The study population comprised school children attending the fifth and sixth grades of primary schools located in the regions of Attica, Etoloakarnania, Thessaloniki and Heraklion. The sampling procedure is fully described elsewhere.^15^ For the purpose of the current analysis, a subsample of 24 obese and 23 normal-weight preadolescent girls, as well as 11 obese and 11 normal-weight preadolescent boys (Table 1) was selected. This subsample was initially used to investigate the effect of polymorphism in the *FTO* gene on genome-wide DNA methylation patterns.^16^

### Dietary assessment

Dietary intake data was obtained for two consecutive weekdays and one weekend day, via morning interviews with the children at the school site using the 24-h recall technique. More specifically, all study participants were asked to describe the type and amount of foods and beverages consumed during the previous day, provided that it was a usual day according to the participant's perception. To improve the accuracy of food descriptions, standard household measures (cups, tablespoons, etc) and food models were used to define amounts. At the end of each interview, the interviewers, who were dietitians rigorously trained to minimize interviewer's effect, reviewed the collected food intake data with the respondent to clarify entries, servings and possible forgotten foods. Food intake data were analyzed using the Nutritionist V diet analysis software (version 2.1, 1999, First Databank, San Bruno, CA, USA), which was extensively amended to include traditional Greek recipes, as described in Food Composition Tables of Greek Cooked Foods and Dishes. Furthermore, the database was updated with nutritional information of processed foods provided by independent research institutes, food companies and fast-food chains.

### DNA methylation profiling

The genome-wide Illumina Infinium HumanMethylation27 BeadChip (Illumina, San Diego, CA, USA), which allows interrogation of 27 578 CpG dinucleotides covering 14 495 genes was applied to determine the methylation profile of genomic DNA isolated and purified from the peripheral whole blood. This chip has been shown to give a reliable and reproducible estimation of the methylation profile on a genomic scale.^17^ First, bisulfite conversion of genomic DNA was performed using the EZ DNA Methylation-Gold Kit (Zymo Research, Irvine, CA, USA) according to the manufacturer's protocol. Briefly, 500 ng of DNA was sodium bisulfite-treated, denatured at 98 °C for 10 min, and bisulfite converted at 64 °C for 2.5 h. After conversion, samples were desulfonated and purified using column preparation. Approximately 200 ng of bisulfate-converted DNA was processed according to the Illumina Infinium Methylation Assay protocol. This assay is based on the conversion of unmethylated cytosine (C) nucleotides into uracil/thymine (T) nucleotides by the bisulfite treatment. The DNA was whole-genome amplified, enzymatically fragmented, precipitated, resuspended, and hybridized overnight at 48 °C to locus-specific oligonucleotide primers on the BeadChip. After hybridization, the C or T nucleotides were detected by single-base primer extension. The fluorescence signals corresponding to the C or T nucleotides were measured from the BeadChips using the Illumina iScan scanner. Phenotypes, raw data and background-corrected normalized DNA methylation data are available through the GEO database (http://www.ncbi.nlm.nih.gov/geo/) with accession numbers GSE27860 for the girls and GSE57484 for the boys.

### Data processing

All downstream data processing and statistical analyses were performed with the statistical software R (www.r-project.org) together with the *lumi*,^18^ *limma*^19^ and *IMA*^20^ packages of the Bioconductor project.

#### Data preprocessing

The fluorescence data were preprocessed using the GenomeStudio 2009.2 (Illumina) software. We used the log_2_ ratio of the intensities of methylated probe *versus* unmethylated probe, also called *M*-value, which is more statistically valid for the differential analysis of methylation levels.^21^

#### Quality control

The data were imported and submitted to quality control using a modified version of the *IMA.methy450PP* function of the *IMA* package. The following CpG sites and samples were removed: the sites with missing *β*-values, the sites with detection *P*-value>0.05, the sites having <75% of samples with detection *P*-value<10^−5^, the samples with missing *β*-values, the samples with detection *P*-value>10^−5^ and the samples having <75% of sites with detection *P*-value<10^−5^. A total of 26 168 probes were included in the analysis, after discarding 328 probes that did not reach the quality control together with 1082 probes from the sex chromosomes.

#### Normalization

Quantile normalization was performed on the *M*-values of all the 26 168 CpG sites using the *lumiMethyN* function of the *lumi* package.

#### Annotation

For better interpretation of the genome-wide methylation patterns, we chose to use the expanded annotation table for the Illumina Infinium HumanMethylation450 BeadChip array generated by Price *et al.*^22^ There are a total of 27 578 loci for 27k array, and 1600 of them are not mapped to 450k array. For those unmapped loci, we kept their original annotation from the 27k array. The expanded annotation file was used to determine the average methylation value of CpG sites belonging to the same island or island shores (all sites with the same name in the ‘HIL_CpG_Island_Name' column of the annotation file were averaged). We obtained the average methylation value of 5980 islands/island shores, which reduced the number of interrogated locations to 19 437 sites/islands. The CpG island classification developed by Price *et al*^22^ provides good enrichment discrimination of CpG islands. This classification is a combination of Weber *et al*'s classification^23^ where CpG islands are defined according to the GC content, the Obs/Exp CpG ratio and the island length, and Illumina's classification, where CpG islands are defined according to their physical position (islands, island shores, and shelves). The location within a CpG island or shore are suggested to be relevant,^24^ and Price *et al*'s definition of CpG islands allowed to distinguish different methylation distribution between probes, which remained undetectable with the Illumina CpG island classification.^22^ Besides, their classification demonstrated a more extreme DNA methylation profile and a larger proportion of differentially methylated regions between different tissues.

The expanded annotation file was also used to determine which gene each interrogated CpG site/island may be associated with (‘Closest_TSS_gene_name' column of the annotation file), the distance of each interrogated CpG site/island to the closest TSS (transcription start site) (‘Distance_closest_TSS' column of the annotation file) and the CpG density surrounding each interrogated CpG site/island (‘HIL_CpG_class' column of the annotation file). Each site can either be located in a high-density CpG island, an intermediate-density CpG island, a region of intermediate-density CpG island that borders HCs, or a non-island. Indeed, the local CpG density has been shown to influence the role of methylated cytosines, with methylation having more transcriptional effect in high-density CpG island and less at non-islands.^25^

The Illumina-provided MAPINFO GenomeStudio column was used to determine the genomic location of each interrogated CpG site. For CpG islands, the name of the island was used to determine its genomic location (eg the island ‘chr19_IC:17905037-17906698' would be a CpG island of intermediate density located on chromosome 19, between 17 905 037 and 17 906 698).

### Statistics

#### Linear model

We developed the following linear model for each CpG site k, using *limma*'s robust regression method, with a maximum number of iteration equal to 10 000:


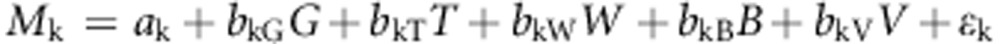


where *M*_k_ is the *M*-value of CpG site/island *k*, *G* is the dichotomized gender (female=1 and male=0), *T* is the Tanner stage, *B* is the white blood cell count, *W* is the dichotomized weight category (normal-weight=0 and obese=1), *ɛ*_k_ is the unexplained variability, and *V* is one of the following variables: proportion of energy intake derived from total fat intake, cholesterol intake (g/day), MUFA/SFA, PUFA/SFA, or (MUFA+PUFA)/SFA.

The coefficients *b*_kx_ summarize the correlation between the methylation level and the variables of interest. Moderated *t*-statistics for each contrast and CpG site/island were created using an empirical Bayes model, to rank genes in order of evidence for differential methylation.^19^ To control the proportion of false positives, *P*-values were adjusted for multiple comparisons as proposed by Benjamini and Hochberg (BH).^26^ An adjusted *P*-value>0.05 was considered nonsignificant.

Three children from the cohort had a MUFA/SFA, a PUFA/SFA, and a (MUFA+PUFA)/SFA higher than the mean±3 × SD. Thus, they were excluded from the linear models developed for MUFA/SFA, PUFA/SFA, and (MUFA+PUFA)/SFA.

#### Functional enrichment analysis

The unique Entrez Gene ID associated with each significant gene-based site/island was identified. Three gene lists were generated for MUFA/SFA, PUFA/SFA, and (MUFA+PUFA)/SFA, respectively.

We used the web-based ConsensusPathDB-human (CPDB)^27, 28^ to determine the significant pathways each gene list may be involved in. On the basis of the reference gene set (all Entrez Gene IDs from the 27k BeadChip annotation file were used as a background), the expected number of genes in each pathway of the CPDB database is compared with the actual number of genes found for this pathway. For each pathway, a *P*-value and a *q*-value are calculated according to the hypergeometric test. The pathways with a raw *P*-value<0.05 together with a *q*-value<0.05 were selected. As CPDB includes information from 30 databases, the pathways often overlap with each other to some extent. Thus, to show the relationships between the different pathways, we constructed a heatmap of the proportion of shared input genes between the significant pathways. For instance, if P1 is a given pathway containing genes *A*, *B*, and *C* from the input gene list, and P2 is a given pathway containing genes *B*, *C*, *D*, and *E* from the input gene list, the proportion of shared genes between P1 and P2 is:


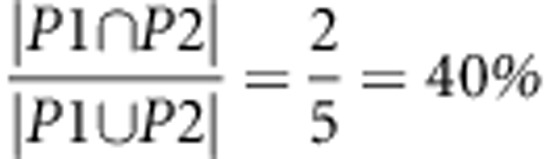


We also used the web-based g:Profiler^29, 30^ as an alternative method for pathway analysis, to confirm the significant results obtained with CPDB. The g:GOSt tool was used for enrichment analysis, with the same background gene list, and the g:GOSt native method g:SCS for multiple testing correction. The pathways with an adjusted *P*-value<0.05 were selected. It is important to note that g:Profiler only includes pathways from two databases: KEGG and Reactome.

## Results

**Figure 1:**
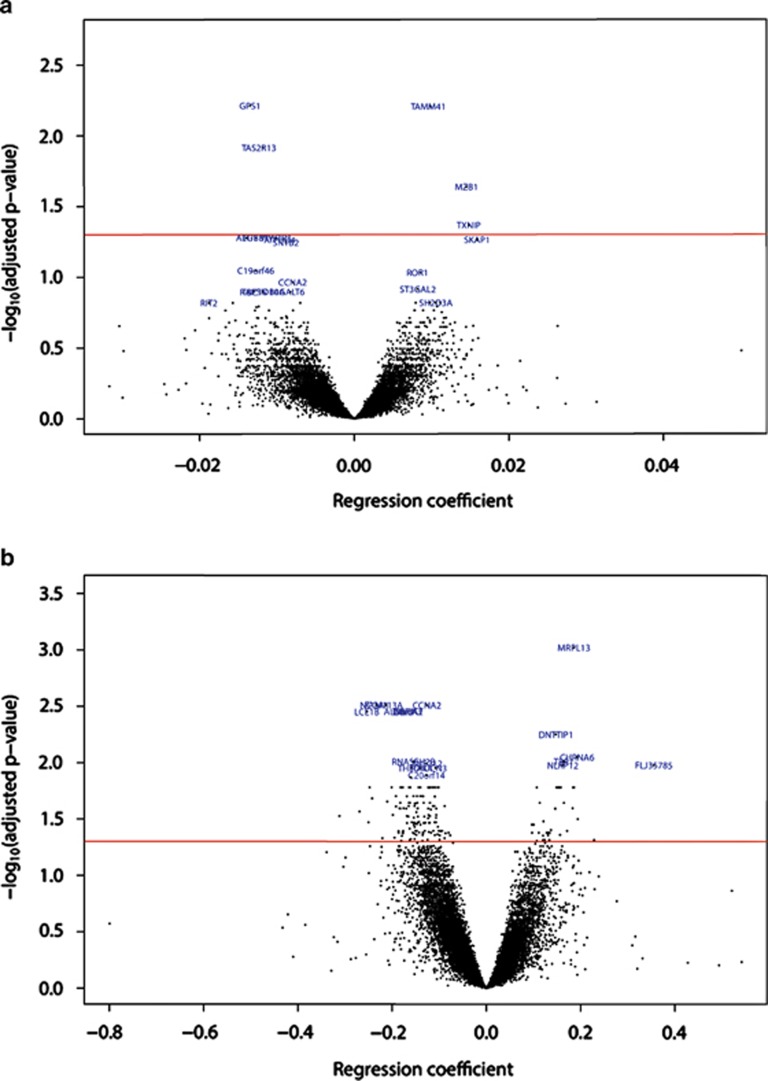
Volcano plots for proportion of total energy intake derived from fat (**a**) and (MUFA+PUFA)/SFA (**b**). The regression coefficient refers to the coefficient of the linear model and each point represents a CpG site or a CpG island. The red horizontal line is the significance threshold (*P*-value=0.05) and all points above this line are significant. (**a**) proportion of total energy intake derived from fat (positive coefficients refer to an increased methylation in children for whom fat represents a higher proportion of total energy intake). (**b**) (MUFA+PUFA)/SFA (positive coefficients refer to an increased methylation in children having a higher (MUFA+PUFA)/SFA).

Four CpG sites and one CpG island were found to be significantly associated with the proportion of overall fat intake (Figure 1a), while no significance was found for cholesterol intake. The methylation levels of 2 islands, 11 island shores, and 16 sites were significantly correlated with PUFA/SFA; 9 islands, 26 island shores, and 158 sites for MUFA/SFA; 10 islands, 40 island shores, and 130 sites for (MUFA+PUFA)/SFA (Figure 1b and Supplementary Tables 1–3).

### What genes are associated with the significant CpG sites/islands?

To determine which gene may be regulated by each CpG site and island, we identified the gene whose TSS is closest to each CpG site and island. Each significant site, island or island shore can show either a positive fold change if its methylation is higher in children having an elevated dietary variable (eg, a higher cholesterol intake), or a negative fold change if its methylation is lower in children having an elevated dietary variable.

Regarding the proportion of fat intake, one CpG site associated with taste receptor, type 2, member 13 (*TAS2R13)* that may have a role in the perception of bitterness, while another site associated with thioredoxin interacting protein (*TXNIP)*, a regulator of cellular oxidative stress downregulated by SFA uptake^31^ (Table 2).

The 10 most significant sites/islands/island shores found for MUFA/SFA included one CpG site associated with aldehyde dehydrogenase 3 family, member A2 (*ALDH3A2)* (*P*=0.00097), whose expression is reduced in insulin-resistant murine models.^32^ It also included a CpG site associated with sema domain, immunoglobulin domain (Ig), short basic domain, secreted, (semaphorin) 3G (*SEMA3G*) (*P*=0.0039), whose expression increases during adipogenesis.^33^ Among the top 10 found for PUFA/SFA, there was 1 CpG site associated with nuclear receptor coactivator 1 (*NCOA1)* (*P*=0.0072) and another 1 associated with PC-esterase domain containing 1A (*PCED1A*) (*P*=0.0091), as well as an island shore associated with phosphodiesterase 3A, cGMP-inhibited (*PDE3A*) (*P*=0.0066; Table 2).

**Figure 2:**
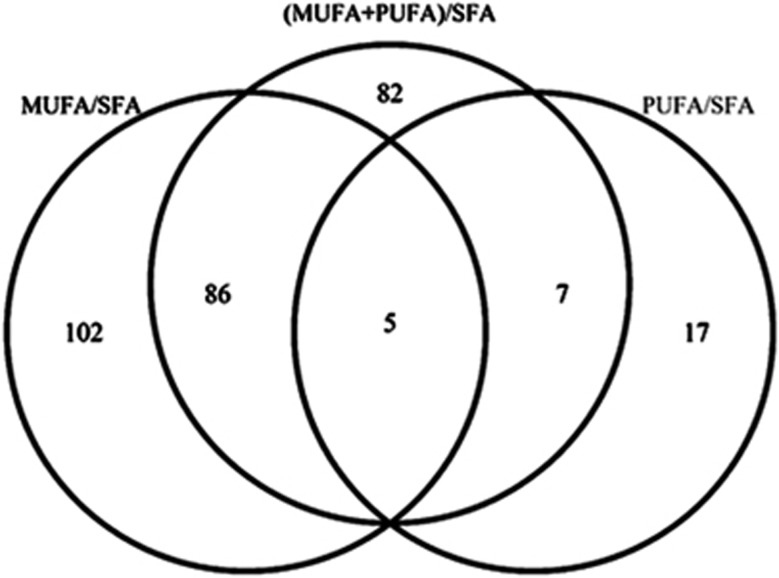
Venn diagram of the significant CpG sites and islands found for MUFA/SFA, PUFA/SFA, and (MUFA+PUFA)/SFA.

**Figure 3:**
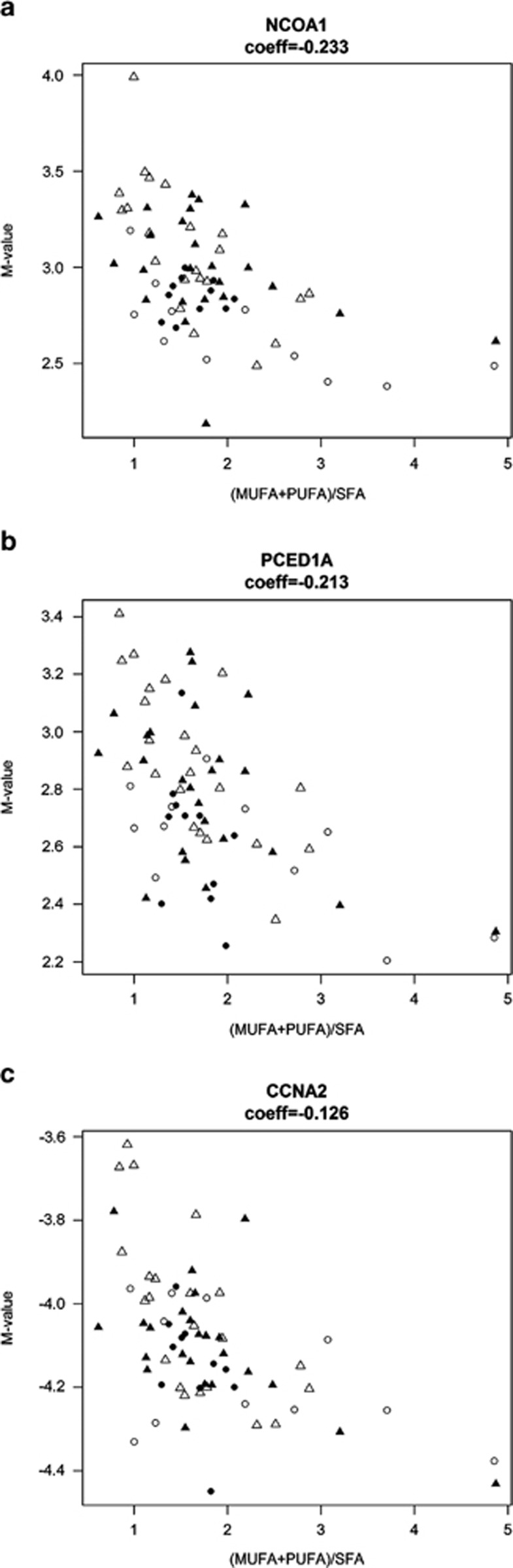
Correlation between methylation of three sites associated with NCOA1 (**a**), PCED1A (**b**), *CCNA2* (**c**), and (MUFA+PUFA)/SFA. Coeff (coefficient) of the linear model associated with (MUFA+PUFA)/SFA; full triangles, obese girls (*n*=23); full circles, obese boys (*n*=11); empty triangles, normal-weight girls (*n*=22); empty circles, normal-weight boys (*n*=10).

There were only 4 sites and 1 island shore found significant for all fatty acid ratios, but 86 sites/islands/island shores in common between MUFA/SFA and (MUFA+PUFA)/SFA, and 7 in common between PUFA/SFA and (MUFA+PUFA)/SFA (Figure 2). Notably, the four sites found significant for all fatty acid ratios contained some of previously mentioned sites (Table 2), for example, the ones associated with *NCOA1* (*P*=0.0031) (Figure 3a) and *PCED1A* (*P*=0.0031) (Figure 3b). It also included an island shore associated with *CCNA2* (Figure 3c), a gene recently shown to be associated with serum phosphatidylcholine concentration in mice.^34^

### In which pathways are the significant genes involved?

Instead of going through all the genes associated with the significant sites found for MUFA/SFA, PUFA/SFA, and (MUFA+PUFA)/SFA, it was preferred to perform a gene enrichment analysis. Using CPDP,^27^ we identified the significant pathways for each of the fatty acid ratios. We considered a pathway significant if the significant CpG sites/island/island shores were associated with a high proportion of genes involved in this particular pathway.

**Figure 4:**
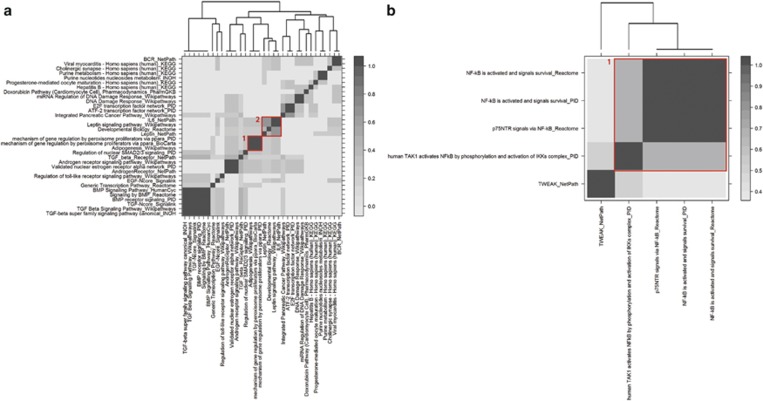
Heatmap representation of the proportion of shared genes between the significant pathways found for PUFA/SFA (**a**) and (MUFA+PUFA)/SFA (**b**). Each significant pathway retrieved from CPDB (*P*-value<0.05 and *q*-value<0.05) is represented on the graph, along with the database it comes from. A stronger color indicates a higher proportion of shared genes between two pathways. (**a**) Red rectangle 1: group of pathways related to adipogenesis and mechanism of gene regulation by peroxisome proliferators *via* PPAR*α*; red rectangle 2: group of pathways related to leptin and IL6. (**b**) Red rectangle 1: group of pathways related to NF-*κ*B. The full colour version of this figure is available at *European Journal of Human Genetics* online.

Neither CPDB nor g:Profiler identified significant pathways for MUFA/SFA, but CPDB found 34 significant pathways for PUFA/SFA (Supplementary Table 4), including 1 group of pathways related to adipogenesis and mechanism of gene regulation by peroxisome proliferators *via* PPAR*α* (Group 1, Figure 4a), and another group of pathways related to leptin and IL6 (Group 2, Figure 4a). Five significant pathways were identified for (MUFA+PUFA)/SFA using CPDB (Supplementary Table 4), including one group of pathways linked to NF-*κ*B (Group 1, Figure 4b). g:Profiler identified only one significant pathway for (MUFA+PUFA)/SFA, also linked to NF-*κ*B (IKK*β* phosphorylates IkB causing NF-*κ*B to dissociate, *P*-value=0.041).

## Discussion

In the present study of Greek preadolescents, we found a large number of CpG sites and regions significantly associated with variables related to the quality of fat intake and few sites significantly associated with variables related to the quantity of fat intake.

Our findings suggest that fat quality is likely to influence DNA methylation on a large genomic scale. *NCOA1*, one of the most significant gene found for all fatty acids ratios, is involved in the mechanism of gene regulation by peroxisome proliferators *via* PPAR*α*, a master gene whose regulation is altered in obesity.^35^ NCOA1 is a transcriptional coactivator whose ablation confers susceptibility to diet-induced obesity.^36^ Interestingly, various fatty acids, but especially PUFAs, act as ligands for PPAR*α*. Moreover, along with *PDE3A,* the fifth most significant gene found for PUFA/SFA, *NCOA1*, is part of the leptin pathway. Leptin is an adipokine that has a key role in regulating energy intake by inhibiting the sensation of hunger.^37^ Fish oil has been reported to increase plasma leptin concentrations,^38^ and leptin induces the expression of *NCOA1* in human cells.^39^ Besides, *PDE3A*'s expression is enhanced in cows fed with a diet enriched in fish oil or in SFA.^40^ Interestingly, an island shore located near the TSS of *PDE3A* was less methylated in children with a higher PUFA/SFA. All this information is consistent with the negative fold change observed for *NCOA1* in our cohort.

There was substantial overlap between the significant sites/islands/island shores found for the different fatty acid ratios, but little overlap between all fatty acid ratios. This may reflect how MUFA and PUFA affect DNA methylation in a different way. Interestingly, the site associated with *NCOA1* was more significant for (MUFA+PUFA)/SFA than for PUFA/SFA or MUFA/SFA, suggesting that PUFA and MUFA affect the methylation of this gene in an identical way. A similar observation can be made for *PCED1A* and *CCNA2* that were more significant for (MUFA+PUFA)/SFA than for PUFA/SFA or MUFA/SFA. However, this may also be due to differences in power to detect significant correlations, as the fatty acids ratios distributions were quite different (Supplementary Figure 1).

It should be noted that two of the four individual CpG sites found to be significantly associated with the proportion of energy intake derived from fat might be relevant to obesity. It has been hypothesized that individuals with increased bitter taste sensitivity avoid antioxidant-rich vegetables because of their perceived bitterness, consuming instead sweet, fatty foods.^41^ The site associated with *TAS2R13* was more methylated in children for whom fat represents a higher proportion of the total energy intake. In addition, children with a higher proportion of energy intake derived from fat had a higher methylation at a site located in an island shore near *TXNIP*, which is consistent with the observed downregulation of *TXNIP* by SFA uptake.^31^ None of these genes were previously reported to be differentially methylated depending on fat intake, probably because the methylation assays of previous studies were limited in scope only addressing key genes.

The present work was not devoid of limitations. First of all, our sample size is limited (*n*=69) and therefore replication is needed to confirm our findings and to allow generalization to larger populations. Second, the fatty acid ratios investigated herein are among the most interesting to compare with respect to health, as their roles are heavily debated and researched. However, other fatty acids not examined in this study may reflect other aspects of the quality of fat intake. For example, unsaturated fatty acids includes *trans* unsaturated fatty acids, which have been demonstrated to have adverse effects on health.^42^ In addition, we did not separate n-3 and n-6 PUFA in our study, but these two fatty acids do not have the same effects; while both n-3 and n-6 PUFA have beneficial effects, an excess of n-6 PUFA can cause health disorders.^43^ DNA methylation was assessed in whole peripheral blood, which is the case for most epigenetic studies focused on nutrition, as peripheral changes may occur in relation to overall energy balance.^44^ However, the methylation pattern observed in blood may not always reflect the pattern in other tissues.^45^ The other weakness of this approach is that DNA methylation can vary by blood cell type, and thus the methylation changes associated with the variables investigated in this study may represent an alteration in blood cell composition, rather than a change in methylation. However, no correlation was found between any of the investigated variables and the relative proportions of granulocytes, lymphocytes, or mid cells (*P*-value>0.05 on Spearman's correlation test). Finally, an increasing number of human studies suggest that parental BMI impacts DNA methylation in the offspring, especially at imprinted genes.^46, 47, 48^ However, evidences in humans are still scarce and limited to two available tissues at birth: umbilical cord and/or placenta; thus, we did not take parental BMI into account in our analysis.

In conclusion, this study is the first to demonstrate the roles of fat quantity and quality in DNA methylation patterns at a genome-wide scale. Our results suggest that specific changes in DNA methylation may have an important role in the mechanisms involved in the physiological responses to different types of dietary fat. Future studies could reveal other potential impacts of dietary fat quality on DNA methylation in controlled, randomized designs, and perhaps investigate further the downstream effects of this process.

## Figures and Tables

**Table 1:** Demographic data stratified for weight category and gender

*Gender*	*Normal-weight*	*Obese*	P*-value*^a^
*Male*			
*N*	11	11	
Age (years)	10.34±0.25	10.82±0.56	0.03
Height (*z*-score)^b^	−1.0±0.20	0.44±0.21	<0.001
Weight (*z*-score)^b^	−0.94±0.093	1.5±0.15	<0.001
BMI (*z*-score)^b^	−0.71±0.11	1.6±0.19	<0.001
White blood cell count (10^3^/mm^3^)	8.56±3.85	6.93±1.27	n.s.
Tanner stage^c^ (*z*-score)^b^	−1.5±0.27	−1.5±0.32	n.s.
Total fat intake (% of total energy intake)	39.92±8.96	45.82±9.54	n.s.
MUFA intake (% of total energy intake)	19.00±5.97	21.74±5.91	n.s.
PUFA intake (% of total energy intake)	7.83±7.99	4.77±0.92	n.s.
SFA intake (% of total energy intake)	13.32±3.62	16.29±3.54	n.s.
Cholesterol (g/day)	188.90±101.84	304.49±137.54	n.s.
			
*Female*			
*N*	23	24	
Age (years)	10.54±0.46	10.94±0.71	0.05
Height (*z*-score)^b^	−0.76±0.16	0.30±0.24	0.001
Weight (*z*-score)^b^	−0.86±0.088	1.5±0.19	<0.001
BMI (*z*-score)^b^	−0.72±0.079	1.8±0.13	<0.001
White blood cell count (10^3^/mm^3^)	7.26±2.07	7.28±1.70	n.s.
Tanner stage^c^ (*z*-score)^b^	−1.45±0.14	−0.70±0.21	n.s.
Total fat intake (% of total energy intake)	42.22±6.59	38.42±7.66	n.s.
MUFA intake (% of total energy intake)	18.83±4.85	17.96±±5.15	n.s.
PUFA intake (% of total energy intake)	7.56±11.42	4.30±2.13	0.04
SFA intake (% of total energy intake)	15.55±2.98	13.67±4.28	0.04
Cholesterol (g/day)	216.25±89.86	211.04±125.58	n.s.

Abbreviations: BMI, body mass index; MUFA, monounsaturated fatty acid; n.s., nonsignificant; PUFA, polyunsaturated fatty acid; SFA, saturated fatty acid.

a Indicates *P*-value for significant or n.s. differences between obese and normal-weight individuals. All values are means±SEs.

b *z*-Scores were calculated using all samples from the Healthy Growth Study as a reference population.

c Describes pubertal development.

**Table 2:** Information on the significant CpG sites/island found for proportion of energy intake derived from fat and the top 10 most significant CpG sites/islands found for MUFA/SFA, PUFA/SFA, and (MUFA+PUFA)/SFA

*Gene*	*Entrez Gene ID*	*Genomic location of the probe/island (hg19)*	*HIL class*^a^	*Genomic location of the closest TSS (hg19)*	*Coefficient*^b^	*Adjusted* P*-value*^c^
*Proportion of energy intake derived from fat*						
GPS1	2873	chr17:80009807	HC	80 009 762	−0.0135	0.00612
TAMM41	13 2001	chr3_HCshore:11887600_11888782; chr3_ICshore:11887684_11888691	HC	11 888 351	0.00987	0.00621
TAS2R13	50 838	chr12:11061985	LC	11 062 160	−0.0118	0.0121
MZB1	51 237	chr5:138725350	LC	138 725 604	0.0145	0.023
TXNIP	10 628	chr1:145438031	IC	145 438 461	0.0148	0.043
						
*MUFA/SFA*						
ALDH3A2	224	chr17:19552343	HC	19 552 063	−0.289	0.00097
MYLK3	91 807	chr16:46782176	LC	46 782 220	−0.238	0.00363
LOC642852	257 103	chr21:46716835	LC	46 707 966	−0.317	0.00364
TPPP2	122 664	chr14:21498837	IC	21 498 344	−0.309	0.00364
RXFP2	122 042	chr13:32313824	NA	32 313 679	−0.262	0.00364
TMEM80	283 232	chr11_HCshore:694282_696564; chr11_ICshore:694282_697179	HC	695 615	−0.245	0.00364
SEMA3G	56 920	chr3:52478874	HC	52 479 042	0.28	0.00388
VCAM1	7412	chr1:101185020	LC	101 185 195	−0.259	0.00482
KRT73	319 101	chr12:53013281	LC	53 012 342	−0.245	0.00496
KRTCAP2	200 185	chr1:155145737	HC	155 145 803	−0.301	0.0051
						
*PUFA/SFA*						
CBR1	873	chr21_HCshore:37441920_37443032; chr21_ICshore:37442016_37442892	HC	37 442 284	1.28	4.02e–06
RBCK1	10 616	chr20:388351	HC	388 708	0.687	2.3e–05
ABHD16A	7920	chr6_HCshore:31670422_31671462; chr6_ICshore:31670279_31671902	HC	31 671 136	−0.302	7.18e–05
KRT23	25 984	chr17:39095141	LC	39 093 835	−0.326	0.00536
PDE3A	5139	chr12_HCshore:20521268_20523183; chr12_ICshore:20520944_20523341	HC	20 522 178	−0.274	0.0066
NCOA1	8648	chr2:24806720	LC	24 807 344	−0.42	0.00722
PCED1A	64 773	chr20:2822804	LC	2 821 796	−0.412	0.00914
MRPL13	27 085	chr8:121457500	HC	121 457 646	0.308	0.0193
AKR7A2	54 896	chr1_HCshore:19638013_19639253; chr1_ICshore:19637904_19639606;	HC	19 638 639	0.237	0.0193
FAM154A	158 297	chr9_IC:19032509_19033364	IC	19 033 255	−0.357	0.0193
						
*(MUFA+PUFA)/SFA*						
MRPL13	27 085	chr8:121457500	HC	121 457 646	0.186	0.000952
NCOA1	8648	chr2:24806720	LC	24 807 344	−0.233	0.00308
PCED1A	64773	chr20:2822804	LC	2821796	−0.213	0.00308
CCNA2	890	chr4_HCshore:122744257_122745486; chr4_ICshore:122744093_122745437	HC	122 745 087	−0.126	0.00308
LCE1B	353 132	chr1:152783674	LC	152 784 446	−0.254	0.00352
ALDH3A2	224	chr17:19552343	HC	19 552 063	−0.176	0.00352
MYLK3	91 807	chr16:46782176	LC	46 782 220	−0.166	0.00352
GBP7	388 646	chr1:89641121	LC	89 641 722	−0.175	0.00352
DGKI	9162	chr7_HCshore:137530917_137532628; chr7_ICshore:137530976_137532560	HC	137 531 608	−0.178	0.00352
DNTTIP1	140 686	chr20:44421526	LC	44 420 575	0.148	0.00561

Abbreviations: HC, high-density CpG island; IC, intermediate-density CpG island; LC, non-island; MUFA, monounsaturated fatty acid; PUFA, polyunsaturated fatty acid; SFA, saturated fatty acid.

a CpG density surrounding each interrogated CpG site/island.

b Value of the coefficient of the linear model associated with (MUFA+PUFA)/SFA.

c *P*-value calculated by moderated t-statistics and adjusted for multiple comparisons according to Benjamini and Hochberg.
