# Supplementary material for: Dietary fat quality impacts genome-wide DNA methylation patterns in a cross-sectional study of Greek preadolescents
Source: Eur J Hum Genet. 2014 Jul 30;23(5):654–62. doi: 10.1038/ejhg.2014.139 (PMC4402618; doi:10.1038/ejhg.2014.139)
Supplement: Supplementary Table 1 [file ejhg2014139x1.doc]

**Additional table 1. Information on the significant CpG sites and islands found for MUFA/SFA.**

| Gene | Entrez Gene ID | Genomic location of the probe/island  (hg19) | HIL class*^1^* | Genomic location of the closest TSS (hg19) | Coefficient*^2^* | Adjusted p-value*^3^* |
| --- | --- | --- | --- | --- | --- | --- |
| ALDH3A2 | 224 | chr17:19552343 | HC | 19552063 | -0.289 | 0.00097 |
| MYLK3 | 91807 | chr16:46782176 | LC | 46782220 | -0.238 | 0.00363 |
| LOC642852 | 257103 | chr21:46716835 | LC | 46707966 | -0.317 | 0.00364 |
| TPPP2 | 122664 | chr14:21498837 | IC | 21498344 | -0.309 | 0.00364 |
| RXFP2 | 122042 | chr13:32313824 | NA | 32313679 | -0.262 | 0.00364 |
| TMEM80 | 283232 | chr11_HCshore:694282_696564;  chr11_ICshore:694282_697179 | HC | 695615 | -0.245 | 0.00364 |
| SEMA3G | 56920 | chr3:52478874 | HC | 52479042 | 0.28 | 0.00388 |
| VCAM1 | 7412 | chr1:101185020 | LC | 101185195 | -0.259 | 0.00482 |
| KRT73 | 319101 | chr12:53013281 | LC | 53012342 | -0.245 | 0.00496 |
| KRTCAP2 | 200185 | chr1:155145737 | HC | 155145803 | -0.301 | 0.0051 |
| COA4 | 80227 | chr11:73586776 | LC | 73587889 | -0.205 | 0.00586 |
| LCE1B | 353132 | chr1:152783674 | LC | 152784446 | -0.363 | 0.00586 |
| DGKI | 9162 | chr7_HCshore:137530917_137532628;  chr7_ICshore:137530976_137532560 | HC | 137531608 | -0.241 | 0.00586 |
| MRPL13 | 27085 | chr8:121457500 | HC | 121457646 | 0.218 | 0.0061 |
| GNG8 | 94235 | chr19:47137948 | NA | 47137939 | 0.229 | 0.0083 |
| TENC1 | 23371 | chr12:53441532 | HC | 53440809 | 0.351 | 0.0083 |
| NLRP12 | 91662 | chr19:54327354 | IC | 54327647 | 0.238 | 0.0083 |
| ARHGAP11A | 9824 | chr15:32907058 | NA | 32907691 | 0.279 | 0.0102 |
| RNASEH2B | 79621 | chr13_HCshore:51483454_51484839;  chr13_ICshore:51483585_51484973 | HC | 51483813 | -0.234 | 0.0112 |
| NGF | 4803 | chr1:115880646 | HC | 115880856 | -0.238 | 0.0118 |
| GBP7 | 388646 | chr1:89641121 | LC | 89641722 | -0.263 | 0.012 |
| POTED | 317754 | chr21:14982175 | HC | 14982497 | -0.373 | 0.0123 |
| UCN3 | 114131 | .;chr10_IC:5406346_5407359 | IC | 5406975 | -0.149 | 0.0123 |
| C1orf85 | 112770 | chr1:156265275 | LC | 156265448 | -0.214 | 0.0137 |
| ST6GALNAC4 | 27090 | chr9:130679027 | HC | 130679304 | -0.31 | 0.0138 |
| SEMA4D | 349236 | chr9:91979776 | LC | 91979565 | -0.325 | 0.0138 |
| TBR1 | 10716 | chr2:162272637 | LC | 162272619 | 0.221 | 0.0138 |
| ZNF623 | 9831 | chr8:144731579 | LC | 144731953 | -0.224 | 0.0138 |
| EFCC1 | 79825 | chr3:128748026 | IC | 128720471 | -0.244 | 0.0138 |
| C2orf47 | 79568 | .;chr2_IC:200818719_200819522 | IC | 200820039 | -0.235 | 0.0138 |
| EYS | 346007 | chr6:66205177 | LC | 66289696 | -0.235 | 0.0146 |
| GSTA5 | 221357 | chr6:52711444 | LC | 52710892 | -0.317 | 0.0146 |
| DNTTIP1 | 140686 | chr20:44421526 | LC | 44420575 | 0.204 | 0.0146 |
| CCNA2 | 890 | chr4_HCshore:122744257_122745486;  chr4_ICshore:122744093_122745437 | HC | 122745087 | -0.174 | 0.0146 |
| SYT3 | 84258 | .;chr19_IC:51141171_51141405 | IC | 51141301 | -0.328 | 0.0146 |
| DEF8 | 54849 | .;chr16_ICshore:90013538_90016268 | ICshore | 90015138 | -0.389 | 0.0146 |
| TRIM60 | 166655 | chr4:165952785 | HC | 165953150 | -0.183 | 0.0181 |
| TMBIM6 | 7009 | chr12:50135197 | NA | 50135385 | -0.175 | 0.0181 |
| DNAH5 | 1767 | chr5:13945990 | LC | 13944588 | -0.248 | 0.0197 |
| COL18A1_AS1 | 378832 | chr21:46844833 | IC | 46844954 | -0.167 | 0.0203 |
| SLC5A12 | 159963 | chr11:26744417 | LC | 26744973 | -0.2 | 0.0209 |
| MTHFD1 | 4522 | chr14_HCshore:64854178_64855310;  chr14_ICshore:64853221_64855611 | HC | 64854758 | -0.161 | 0.0209 |
| LRRC7 | 57554 | chr1:70225863 | LC | 70225857 | 0.164 | 0.0212 |
| CCDC77 | 5927 | chr12:498428 | HC | 498515 | 0.195 | 0.0212 |
| VEPH1 | 79674 | chr3:157217236 | IC | 157217444 | -0.234 | 0.0212 |
| SLC6A15 | 55117 | chr12_HCshore:85306267_85307063;  chr12_ICshore:85305034_85307344 | HC | 85306605 | -0.189 | 0.0212 |
| GNAI1 | 2770 | chr7_HCshore:79763641_79765316;  chr7_ICshore:79763625_79765459 | HC | 79764139 | 0.15 | 0.0212 |
| MOCS3 | 8813 | .;chr20_ICshore:49574317_49576591 | ICshore | 49575362 | -0.242 | 0.0213 |
| TSTD1 | 50848 | chr1:161009703 | LC | 161008773 | -0.184 | 0.022 |
| PCED1A | 64773 | chr20:2822804 | LC | 2821796 | -0.265 | 0.0221 |
| ABCB4 | 5244 | chr7:87109320 | LC | 87105018 | -0.203 | 0.0224 |
| LONP1 | 257062 | chr19_HCshore:5719315_5721498;  chr19_ICshore:5719244_5721592 | HC | 5720462 | -0.165 | 0.0224 |
| GALE | 2582 | chr1:24127833 | NA | 24127294 | 0.234 | 0.0227 |
| ARHGAP32 | 9743 | chr11:128893801 | LC | 128894087 | -0.25 | 0.0231 |
| NUP43 | 348995 | chr6_HCshore:150067237_150067910;  chr6_ICshore:150067214_150067911 | HC | 150067687 | -0.133 | 0.0231 |
| DCST1 | 127579 | chr1:155007204 | IC | 155006281 | -0.27 | 0.0233 |
| CRIP1 | 1396 | chr14:105953642 | NA | 105953549 | -0.208 | 0.0233 |
| CDH10 | 1008 | chr5:24644893 | NA | 24644911 | -0.27 | 0.0233 |
| LARP6 | 55323 | chr15:71146184 | HC | 71146497 | -0.208 | 0.0233 |
| TNS4 | 84951 | chr17:38657260 | LC | 38657853 | -0.241 | 0.0233 |
| RRAGA | 10670 | chr9:19049789 | HC | 19049371 | 0.163 | 0.0233 |
| WWTR1 | 25937 | chr3_HCshore:149374479_149376047;  chr3_ICshore:149374415_149376867 | HC | 149375811 | -0.181 | 0.0233 |
| BCAS4 | 55653 | chr20_HCshore:49410694_49412436;  chr20_ICshore:49410221_49412296 | HC | 49411466 | -0.201 | 0.0233 |
| KCNK7 | 10089 | chr11:65362899 | LC | 65363466 | -0.183 | 0.0242 |
| KCNJ14 | 3770 | chr19:48958664 | LC | 48958765 | -0.196 | 0.0242 |
| OTUD7A | 161725 | chr15:31947546 | NA | 151298724 | -0.26 | 0.0242 |
| YIF1A | 10897 | chr11:66056894 | HC | 66056637 | -0.204 | 0.0244 |
| DNAJC14 | 85406 | chr12:56224078 | ICshore | 56224341 | -0.163 | 0.0244 |
| LPPR4 | 9890 | .;chr1_IC:99729435_99730742 | IC | 99729847 | 0.15 | 0.0244 |
| XBP1 | 7494 | chr22:29196047 | HC | 29196559 | 0.225 | 0.0244 |
| ZNF259 | 8882 | chr11:116659079 | HC | 116658738 | 0.206 | 0.0244 |
| BPIFB4 | 149954 | chr20:31670562 | LC | 31669317 | -0.234 | 0.0248 |
| GATAD2A | 54815 | chr19:19569574 | LC | 19576153 | -0.278 | 0.0248 |
| ABCA4 | 24 | chr1:94587872 | LC | 94586704 | -0.261 | 0.0248 |
| HTRA3 | 94031 | chr4:8270745 | ICshore | 8271488 | -0.194 | 0.0248 |
| OSBPL5 | 114879 | .;chr11_IC:3120882_3122050 | IC | 3116108 | -0.211 | 0.0248 |
| BC035195 | 5494 | chr14:60711647 | LC | 60712367 | -0.205 | 0.0252 |
| NCOA7 | 135112 | chr6:126101898 | IC | 126102306 | 0.246 | 0.0254 |
| SLA2 | 84174 | chr20:35274665 | IC | 35274618 | 0.264 | 0.0254 |
| TMEM180 | 79847 | chr10:104220950 | HC | 104221169 | -0.213 | 0.0254 |
| BACH1 | 571 | chr21:30670416 | ICshore | 30671219 | -0.286 | 0.0254 |
| HMGCS2 | 3158 | chr1:120311599 | LC | 120311554 | -0.233 | 0.0254 |
| NCOA1 | 8648 | chr2:24806720 | LC | 24807344 | -0.306 | 0.0254 |
| PI4KB | 5298 | chr1:151298666 | NA | 151298724 | -0.149 | 0.0254 |
| KIR2DL1 | 3802 | chr19:55281253 | NA | 55281280 | -0.22 | 0.0254 |
| SLC25A2 | 83884 | chr5_HCshore:140683137_140684229;  chr5_ICshore:140683010_140684617 | HC | 140683611 | -0.207 | 0.0254 |
| KANK4 | 163782 | chr1:62785462 | ICshore | 62785082 | -0.221 | 0.0256 |
| ALPK3 | 57538 | chr15:85360217 | ICshore | 85359910 | -0.308 | 0.0256 |
| TRAF3 | 7187 | chr14_HCshore:103242844_103245060;  chr14_ICshore:103242850_103244878 | HC | 103243815 | -0.174 | 0.027 |
| OR2V2 | 285659 | chr5:180581900 | LC | 180581942 | -0.211 | 0.0272 |
| B3GALT5 | 10317 | chr21:41029450 | LC | 41029253 | -0.228 | 0.0272 |
| RPL14 | 9045 | chr3:40499165 | HC | 40498829 | 0.317 | 0.0272 |
| C20orf144 | 128864 | chr20:32250019 | LC | 32250091 | -0.148 | 0.0272 |
| IL22RA2 | 116379 | chr6:137494988 | NA | 137494785 | -0.27 | 0.0272 |
| PLTP | 5360 | chr20:44540621 | NA | 44540786 | 0.189 | 0.0272 |
| CHST12 | 55501 | chr7:2472314 | HC | 2472274 | 0.297 | 0.0272 |
| KIAA1737 | 85457 | chr14_HCshore:77563707_77565064;  chr14_ICshore:77563882_77565146 | HC | 77564577 | -0.136 | 0.0272 |
| MFRP | 83552 | chr11:119217176 | NA | 119217370 | -0.367 | 0.0278 |
| ZBTB49 | 55646 | chr4:4293029 | LC | 4291923 | -0.382 | 0.028 |
| OR2K2 | 26248 | chr9:114090211 | IC | 114090712 | -0.234 | 0.028 |
| FBXL8 | 55336 | chr16:67194079 | HC | 67193890 | -0.18 | 0.028 |
| PGBD4 | 56851 | chr15:34395546 | LC | 34394273 | -0.235 | 0.028 |
| CYP17A1 | 1586 | chr10:104596890 | LC | 104597289 | -0.201 | 0.0287 |
| SLC7A10 | 56301 | chr19:33716273 | HC | 33716755 | 0.238 | 0.0294 |
| FAM135B | 51059 | chr8:139164635 | IC | 139165458 | -0.217 | 0.0294 |
| SSH2 | 85464 | chr17:28256974 | HC | 28257017 | -0.204 | 0.0294 |
| WHSC1 | 7468 | chr4:1893257 | IC | 1894508 | -0.209 | 0.0297 |
| IKBKB | 3551 | chr8_HCshore:42128412_42129300;  chr8_ICshore:42128371_42129504 | HC | 42128819 | -0.153 | 0.0305 |
| CLSPN | 63967 | chr1:36235882 | ICshore | 36235550 | 0.159 | 0.0309 |
| ARHGEF12 | 23365 | chr11:120207730 | HC | 120207617 | -0.189 | 0.0336 |
| MSMB | 4477 | chr10:51549260 | LC | 51549552 | -0.218 | 0.0354 |
| TCIRG1 | 10312 | chr11:67806668 | IC | 67806461 | -0.168 | 0.0354 |
| ARFGAP2 | 84364 | chr11_HCshore:47197898_47199152;  chr11_ICshore:47197677_47199086 | HC | 47198675 | -0.168 | 0.0358 |
| PSTPIP2 | 9050 | chr18_HCshore:43651581_43652812;  chr18_ICshore:43651529_43652853 | HC | 43652249 | -0.145 | 0.0358 |
| SEMG2 | 6407 | chr20:43850461 | LC | 43850009 | -0.196 | 0.0377 |
| LRRC37A | 9884 | chr17:44372704 | NA | 44372497 | -0.233 | 0.0377 |
| MBP | 4155 | chr18:74844766 | HC | 74844773 | -0.223 | 0.0378 |
| MYL7 | 58498 | chr7:44181741 | LC | 44180915 | -0.2 | 0.0383 |
| SCML4 | 256380 | chr6:108093179 | LC | 108093589 | -0.288 | 0.0383 |
| MSLN | 10232 | chr16:811347 | LC | 811072 | -0.243 | 0.0383 |
| ASPA | 443 | chr17:3378347 | LC | 3379295 | -0.192 | 0.0383 |
| SPSB1 | 80176 | chr1:9353265 | HC | 9352940 | -0.19 | 0.0383 |
| PRRC1 | 133619 | chr5_HCshore:126852789_126853860;  chr5_ICshore:126852977_126854140 | HC | 126853308 | 0.217 | 0.0383 |
| STXBP6 | 29091 | chr14:25518574 | HC | 25519094 | 0.158 | 0.0387 |
| SSTR3 | 6753 | chr22:37607676 | IC | 37608352 | -0.243 | 0.0387 |
| CILP | 8483 | chr15:65503447 | LC | 65503839 | -0.199 | 0.0387 |
| HIST1H4G | 8369 | .;chr6_IC:26246750_26247294 | IC | 26247204 | -0.231 | 0.0387 |
| UPK2 | 7379 | chr11:118827082 | LC | 118827007 | -0.189 | 0.039 |
| CHERP | 10523 | chr19:16653599 | NA | 16653248 | 0.206 | 0.039 |
| SERAC1 | 84947 | chr6:158589306 | HC | 158589311 | 0.279 | 0.039 |
| APCS | 325 | chr1:159557836 | LC | 159557615 | -0.185 | 0.039 |
| C19orf80 | 55908 | chr19:11348389 | IC | 11348125 | -0.243 | 0.0392 |
| GBP2 | 2634 | chr1:89591719 | LC | 89591798 | 0.236 | 0.0401 |
| PRDM11 | 56981 | chr11:45115478 | LC | 45115563 | -0.193 | 0.0416 |
| PRND | 23627 | chr20:4702531 | LC | 4702555 | -0.193 | 0.0416 |
| ASB17 | 127247 | chr1:76397797 | LC | 76398115 | -0.16 | 0.0416 |
| NAA16 | 79612 | chr13:41884494 | IC | 41885340 | -0.191 | 0.0416 |
| PMS2 | 5395 | chr7:6048614 | HC | 6048736 | -0.207 | 0.0416 |
| VAV2 | 7410 | chr9:136858680 | ICshore | 136857445 | 0.167 | 0.0416 |
| SHC1 | 1163 | chr1:154946946 | HC | 154946958 | -0.166 | 0.0426 |
| ORAI2 | 80228 | chr7:102073539 | ICshore | 102073995 | 0.335 | 0.0426 |
| C9orf24 | 84688 | chr9:34397651 | LC | 34397848 | -0.203 | 0.0426 |
| SF1 | 7536 | chr11_HCshore:64544821_64546797;  chr11_ICshore:64544940_64546776 | HC | 64546315 | -0.149 | 0.0426 |
| CSMD1 | 64478 | chr8:4853275 | LC | 4852327 | -0.252 | 0.0428 |
| TMOD4 | 29765 | chr1:151148266 | LC | 151148456 | -0.206 | 0.0428 |
| TMTC4 | 84899 | chr13:101295638 | IC | 101294564 | -0.259 | 0.0428 |
| GFM2 | 84340 | chr5:74062846 | HC | 74063041 | 0.255 | 0.0428 |
| FGFR4 | 2264 | chr5:176514203 | HC | 176513920 | 0.156 | 0.0428 |
| CCL25 | 6370 | chr19:8117966 | LC | 8117883 | -0.322 | 0.0428 |
| FOXC2 | 2303 | chr16:86600389 | HC | 86600856 | -0.234 | 0.0428 |
| PDHA2 | 5161 | chr4:96761327 | HC | 96761238 | -0.217 | 0.0428 |
| HIP1 | 3092 | chr7_HCshore:75367845_75369172;  chr7_ICshore:75367652_75370056 | HC | 75368282 | 0.134 | 0.0428 |
| NR0B2 | 8431 | .;chr1_IC:27240168_27240377 | IC | 27240566 | -0.219 | 0.0428 |
| HIST1H2AI | 8329 | chr6:27776308 | NA | 27775977 | -0.229 | 0.0432 |
| ZNF432 | 9668 | chr19:52551568 | LC | 52552072 | 0.35 | 0.0432 |
| PRDX6 | 9588 | chr1:173446965 | NA | 173446486 | -0.168 | 0.0432 |
| KCNQ2 | 3785 | chr20:62103578 | HC | 62103992 | -0.212 | 0.0433 |
| SPAST | 6683 | chr2:32288253 | HC | 32288679 | 0.196 | 0.0433 |
| CNTFR | 1271 | chr9:34589381 | HC | 34589734 | -0.219 | 0.0433 |
| SHC3 | 53358 | chr9:91793648 | HC | 91793681 | -0.175 | 0.0433 |
| RAD54B | 25788 | chr8:95487224 | NA | 95487310 | 0.203 | 0.0433 |
| KBTBD4 | 4722 | chr11:47600628 | HC | 47600566 | -0.146 | 0.0433 |
| CAB39 | 51719 | chr2:231578417 | HC | 231578262 | -0.185 | 0.0433 |
| BAG4 | 27257 | chr8:38033835 | HC | 38034105 | 0.25 | 0.0433 |
| IRAK4 | 83448 | chr12:44153803 | LC | 44152746 | -0.18 | 0.0433 |
| UHRF2 | 115426 | chr9_HCshore:6411902_6414489;  chr9_ICshore:6411820_6414388 | HC | 6413150 | -0.143 | 0.0433 |
| DCUN1D1 | 54165 | chr3:182697846 | HC | 182698325 | -0.268 | 0.0433 |
| HSPB3 | 8988 | chr5:53751361 | LC | 53751430 | -0.189 | 0.0445 |
| STAU1 | 6780 | chr20:47804099 | NA | 47804904 | 0.183 | 0.0446 |
| HEPACAM2 | 253012 | chr7:92849851 | LC | 92848869 | -0.266 | 0.0446 |
| NEU2 | 4759 | chr2:233896069 | LC | 233897381 | -0.204 | 0.0446 |
| LACTB | 114294 | chr15:63414018 | HC | 63413998 | -0.234 | 0.0446 |
| SMAD9 | 4093 | .;chr13_IC:37453219_37454095 | IC | 37454011 | -0.133 | 0.0446 |
| GOLGA8I | 283796 | chr15:23255338 | NA | 23255242 | 0.371 | 0.0448 |
| C5 | 727 | chr9:123811564 | LC | 123812553 | -0.22 | 0.045 |
| PEX11G | 92960 | chr19:7553274 | ICshore | 7553904 | -0.238 | 0.045 |
| RPS13 | 6207 | chr11:17098772 | NA | 17099220 | -0.148 | 0.045 |
| TIMD4 | 91937 | chr5:156389649 | LC | 156390265 | -0.197 | 0.045 |
| UBE2F | 140739 | chr2:238876345 | HC | 238875586 | -0.198 | 0.045 |
| ZNF322 | 79692 | chr6:26659487 | HC | 26659979 | 0.201 | 0.045 |
| CCBP2 | 1238 | chr3:42850848 | LC | 42850964 | -0.26 | 0.0454 |
| S100A11 | 6282 | chr1:152009284 | ICshore | 152009510 | 0.196 | 0.0454 |
| IGHG1 | 3500 | chr14:106209207 | NA | 106209407 | -0.204 | 0.0454 |
| PRSS21 | 10942 | chr16:2867088 | NA | 2867164 | -0.243 | 0.0455 |
| RNF103 | 7844 | chr2:86851318 | HC | 86850999 | 0.195 | 0.0466 |
| PAICS | 10606 | chr4_HCshore:57301267_57303402;  chr4_ICshore:57300926_57304017 | HC | 57301917 | 0.211 | 0.0466 |
| CEBPD | 1052 | chr8_HCshore:48649521_48651940;  chr8_ICshore:48649141_48651803 | HC | 48650725 | -0.152 | 0.0466 |
| HERC4 | 26091 | chr10:69835287 | ICshore | 69835102 | 0.297 | 0.0468 |
| BRP44L | 51660 | chr6_HCshore:166795170_166797534;  chr6_ICshore:166795266_166798090 | HC | 166796485 | -0.173 | 0.0468 |
| SLC5A4 | 6527 | chr22:32650997 | LC | 32651317 | -0.254 | 0.047 |
| NUMA1 | 4926 | chr11:71793001 | LC | 71791572 | -0.158 | 0.0484 |
| AQP12A | 375318 | chr2:241631448 | IC | 241631261 | 0.297 | 0.0488 |
| ORAOV1 | 595 | .;chr11_IC:69467820_69470023 | IC | 69469177 | -0.141 | 0.0488 |

*^1^*CpG density surrounding each interrogated CpG site/island. HC, high-density CpG island; IC, intermediate-density CpG island; ICshore, IC that overlaps with some HC; LC, non-island.

*^2^*Value of the coefficient of the linear model associated with MUFA/SFA

*^3^*P-value calculated by moderated t-statistics and adjusted for multiple comparisons according to Benjamini and Hochberg.
