# Supplementary material for: Dietary fat quality impacts genome-wide DNA methylation patterns in a cross-sectional study of Greek preadolescents
Source: Eur J Hum Genet. 2014 Jul 30;23(5):654–62. doi: 10.1038/ejhg.2014.139 (PMC4402618; doi:10.1038/ejhg.2014.139)
Supplement: Supplementary Table 2 [file ejhg2014139x2.doc]

**Additional table 2. Information on the significant CpG sites and islands found for PUFA/SFA.**

| Gene | Entrez Gene ID | Genomic location of the probe/island  (hg19) | HIL class*^1^* | Genomic location of the closest TSS (hg19) | Coefficient*^2^* | Adjusted p-value*^3^* |
| --- | --- | --- | --- | --- | --- | --- |
| CBR1 | 873 | chr21_HCshore:37441920_37443032;  chr21_ICshore:37442016_37442892 | HC | 37442284 | 1.28 | 4.02e-06 |
| RBCK1 | 10616 | chr20:388351 | HC | 388708 | 0.687 | 2.3e-05 |
| ABHD16A | 7920 | chr6_HCshore:31670422_31671462;  chr6_ICshore:31670279_31671902 | HC | 31671136 | -0.302 | 7.18e-05 |
| KRT23 | 25984 | chr17:39095141 | LC | 39093835 | -0.326 | 0.00536 |
| PDE3A | 5139 | chr12_HCshore:20521268_20523183;  chr12_ICshore:20520944_20523341 | HC | 20522178 | -0.274 | 0.0066 |
| NCOA1 | 8648 | chr2:24806720 | LC | 24807344 | -0.42 | 0.00722 |
| PCED1A | 64773 | chr20:2822804 | LC | 2821796 | -0.412 | 0.00914 |
| MRPL13 | 27085 | chr8:121457500 | HC | 121457646 | 0.308 | 0.0193 |
| AKR7A2 | 54896 | chr1_HCshore:19638013_19639253;  chr1_ICshore:19637904_19639606 | HC | 19638639 | 0.237 | 0.0193 |
| FAM154A | 158297 | .;chr9_IC:19032509_19033364 | IC | 19033255 | -0.357 | 0.0193 |
| SKI | 6497 | chr1:2159324 | HC | 2160133 | 0.367 | 0.0217 |
| PDZD2 | 23037 | chr5:31638901 | HC | 31639516 | -0.335 | 0.0217 |
| CLEC12B | 387837 | chr12:10161879 | NA | 10163231 | -0.332 | 0.0238 |
| CHRNA6 | 8973 | chr8:42623896 | LC | 42623928 | 0.38 | 0.0242 |
| CYCS | 54205 | chr7:25164393 | HC | 25164979 | 0.385 | 0.0242 |
| SCN2A | 6326 | chr2:166150400 | NA | 166150592 | -0.444 | 0.0293 |
| LARP1B | 55132 | chr4_HCshore:128981633_128983684;  chr4_ICshore:128981733_128983779 | HC | 128982502 | -0.242 | 0.0293 |
| FYN | 2534 | chr6_HCshore:112193652_112195076;  chr6_ICshore:112193585_112195179 | HC | 112194654 | 0.182 | 0.0293 |
| ZDHHC11 | 79844 | chr5:851372 | IC | 851100 | -0.436 | 0.0329 |
| ZNF212 | 7988 | chr7_HCshore:148936414_148937478;  chr7_ICshore:148936437_148937685 | HC | 148936741 | -0.254 | 0.0329 |
| NBR2 | 672 | chr17_HCshore:41278108_41278811;  chr17_ICshore:41277969_41279365 | HC | 41277599 | -0.347 | 0.0356 |
| CCNA2 | 890 | chr4_HCshore:122744257_122745486;  chr4_ICshore:122744093_122745437 | HC | 122745087 | -0.228 | 0.0388 |
| SMAD6 | 4091 | chr15_HCshore:66992836_66996736;  chr15_ICshore:66992799_66996718 | HC | 66994673 | 0.158 | 0.0388 |
| NR2F1 | 7025 | chr5:92918517 | IC | 92919042 | 0.27 | 0.0436 |
| HCFC1R1 | 54985 | chr16_HCshore:3073791_3074709;  chr16_ICshore:3073568_3074905 | HC | 3074286 | -0.264 | 0.0436 |
| PRPS1L1 | 221823 | chr7:18067463 | IC | 18067485 | -0.393 | 0.0447 |
| SPAST | 6683 | chr2:32288253 | HC | 32288679 | 0.324 | 0.0447 |
| KIAA0040 | 9674 | chr1:175162044 | HC | 175161948 | 0.614 | 0.0447 |
| B3GNT3 | 10331 | .;chr19_IC:17905037_17906698 | IC | 17905918 | -0.44 | 0.0447 |

*^1^*CpG density surrounding each interrogated CpG site/island. HC, high-density CpG island; IC, intermediate-density CpG island; LC, non-island.

*^2^*Value of the coefficient of the linear model associated with PUFA/SFA

*^3^*P-value calculated by moderated t-statistics and adjusted for multiple comparisons according to Benjamini and Hochberg.
