# Supplementary material for: Dietary fat quality impacts genome-wide DNA methylation patterns in a cross-sectional study of Greek preadolescents
Source: Eur J Hum Genet. 2014 Jul 30;23(5):654–62. doi: 10.1038/ejhg.2014.139 (PMC4402618; doi:10.1038/ejhg.2014.139)
Supplement: Supplementary Table 3 [file ejhg2014139x3.doc]

**Additional table 3. Information on the significant CpG sites and islands found for (MUFA+PUFA)/SFA.**

| Gene | Entrez Gene ID | Genomic location of the probe/island  (hg19) | HIL class*^1^* | Genomic location of the closest TSS (hg19) | Coefficient*^2^* | Adjusted p-value*^3^* |
| --- | --- | --- | --- | --- | --- | --- |
| MRPL13 | 27085 | chr8:121457500 | HC | 121457646 | 0.186 | 0.000952 |
| NCOA1 | 8648 | chr2:24806720 | LC | 24807344 | -0.233 | 0.00308 |
| PCED1A | 64773 | chr20:2822804 | LC | 2821796 | -0.213 | 0.00308 |
| CCNA2 | 890 | chr4_HCshore:122744257_122745486;  chr4_ICshore:122744093_122745437 | HC | 122745087 | -0.126 | 0.00308 |
| LCE1B | 353132 | chr1:152783674 | LC | 152784446 | -0.254 | 0.00352 |
| ALDH3A2 | 224 | chr17:19552343 | HC | 19552063 | -0.176 | 0.00352 |
| MYLK3 | 91807 | chr16:46782176 | LC | 46782220 | -0.166 | 0.00352 |
| GBP7 | 388646 | chr1:89641121 | LC | 89641722 | -0.175 | 0.00352 |
| DGKI | 9162 | chr7_HCshore:137530917_137532628;  chr7_ICshore:137530976_137532560 | HC | 137531608 | -0.178 | 0.00352 |
| DNTTIP1 | 140686 | chr20:44421526 | LC | 44420575 | 0.148 | 0.00561 |
| CHRNA6 | 8973 | chr8:42623896 | LC | 42623928 | 0.192 | 0.00893 |
| TBR1 | 10716 | chr2:162272637 | LC | 162272619 | 0.164 | 0.00976 |
| RNASEH2B | 79621 | chr13_HCshore:51483454_51484839;  chr13_ICshore:51483585_51484973 | HC | 51483813 | -0.155 | 0.00976 |
| ZNF212 | 7988 | chr7_HCshore:148936414_148937478;  chr7_ICshore:148936437_148937685 | HC | 148936741 | -0.127 | 0.0101 |
| NLRP12 | 91662 | chr19:54327354 | IC | 54327647 | 0.162 | 0.0105 |
| GOLGA8I | 283796 | chr15:23255338 | NA | 23255242 | 0.355 | 0.0105 |
| THSD4 | 79875 | chr15:72020908 | IC | 72050171 | -0.159 | 0.0112 |
| IRAK4 | 83448 | chr12:44153803 | LC | 44152746 | -0.139 | 0.0112 |
| UCN3 | 114131 | .;chr10_IC:5406346_5407359 | IC | 5406975 | -0.108 | 0.0112 |
| C20orf144 | 128864 | chr20:32250019 | LC | 32250091 | -0.127 | 0.0129 |
| EYS | 346007 | chr6:66205177 | LC | 66289696 | -0.16 | 0.0134 |
| SLA2 | 84174 | chr20:35274665 | IC | 35274618 | 0.186 | 0.0165 |
| CRIP1 | 1396 | chr14:105953642 | NA | 105953549 | -0.163 | 0.0165 |
| GNG8 | 94235 | chr19:47137948 | NA | 47137939 | 0.154 | 0.0165 |
| SEMA3G | 56920 | chr3:52478874 | HC | 52479042 | 0.184 | 0.0165 |
| KRT73 | 319101 | chr12:53013281 | LC | 53012342 | -0.14 | 0.0165 |
| CLEC12B | 387837 | chr12:10161879 | NA | 10163231 | -0.144 | 0.0165 |
| SPAST | 6683 | chr2:32288253 | HC | 32288679 | 0.152 | 0.0165 |
| DNAJC14 | 85406 | chr12:56224078 | ICshore | 56224341 | -0.123 | 0.0165 |
| TPPP2 | 122664 | chr14:21498837 | IC | 21498344 | -0.201 | 0.0165 |
| MOG | 4340 | chr6:29624796 | LC | 29624757 | -0.164 | 0.0165 |
| P2RX6 | 9127 | chr22:21369560 | LC | 21369475 | -0.177 | 0.0165 |
| ZNF259 | 8882 | chr11:116659079 | HC | 116658738 | 0.156 | 0.0165 |
| CCL25 | 6370 | chr19:8117966 | LC | 8117883 | -0.248 | 0.0165 |
| ZNF322 | 79692 | chr6:26659487 | HC | 26659979 | 0.149 | 0.0165 |
| KRTCAP2 | 200185 | chr1:155145737 | HC | 155145803 | -0.182 | 0.0165 |
| ORAOV1 | 220064 | .;chr11_IC:69467820_69470023 | IC | 69469177 | -0.101 | 0.0165 |
| C20orf112 | 140688 | chr20_HCshore:31070089_31072961;  chr20_ICshore:31070018_31072883 | HC | 31071287 | -0.121 | 0.0165 |
| ANAPC11 | 51529 | chr17_HCshore:79848377_79850296;  chr17_ICshore:79848338_79850676 | HC | 79849817 | -0.103 | 0.0165 |
| DNAJC28 | 54943 | chr21_HCshore:34863559_34864432;  chr21_ICshore:34863510_34864453 | HC | 34863789 | -0.113 | 0.0165 |
| PAICS | 10606 | chr4_HCshore:57301267_57303402;  chr4_ICshore:57300926_57304017 | HC | 57301917 | 0.158 | 0.0165 |
| RAD51 | 5888 | chr15_HCshore:40986464_40988098;  chr15_ICshore:40986173_40988710 | HC | 40987377 | -0.109 | 0.0165 |
| LPPR4 | 9890 | .;chr1_IC:99729435_99730742 | IC | 99729847 | 0.107 | 0.0165 |
| MTHFD1 | 4522 | chr14_HCshore:64854178_64855310;  chr14_ICshore:64853221_64855611 | HC | 64854758 | -0.114 | 0.0165 |
| EDC4 | 23644 | chr16_HCshore:67906650_67907612;  chr16_ICshore:67906652_67907836 | HC | 67907274 | -0.119 | 0.0165 |
| TMEM80 | 283232 | chr11_HCshore:694282_696564;  chr11_ICshore:694282_697179 | HC | 695615 | -0.161 | 0.0165 |
| HIST1H4G | 8369 | .;chr6_IC:26246750_26247294 | IC | 26247204 | -0.179 | 0.0165 |
| TRIM60 | 166655 | chr4:165952785 | HC | 165953150 | -0.122 | 0.0166 |
| ABCB4 | 5244 | chr7:87109320 | LC | 87105018 | -0.136 | 0.0198 |
| PRND | 23627 | chr20:4702531 | LC | 4702555 | -0.155 | 0.0198 |
| C1orf85 | 112770 | chr1:156265275 | LC | 156265448 | -0.14 | 0.0198 |
| IL22RA2 | 116379 | chr6:137494988 | NA | 137494785 | -0.192 | 0.0198 |
| SLC25A2 | 83884 | chr5_HCshore:140683137_140684229;  chr5_ICshore:140683010_140684617 | HC | 140683611 | -0.147 | 0.0198 |
| PZP | 5858 | chr12:9360854 | LC | 9360965 | -0.169 | 0.0199 |
| C4B | 721 | chr6:31948523 | NA | 31949834 | -0.134 | 0.0199 |
| DMRT3 | 58524 | chr9:975856 | ICshore | 976963 | -0.109 | 0.0199 |
| GINS2 | 51659 | chr16:85723489 | ICshore | 85722587 | -0.243 | 0.0207 |
| DCUN1D1 | 54165 | chr3:182697846 | HC | 182698325 | -0.21 | 0.0221 |
| COA4 | 51287 | chr11:73586776 | LC | 73587889 | -0.133 | 0.0227 |
| GRIP1 | 23426 | chr12:67073022 | LC | 67072924 | -0.163 | 0.0227 |
| NCOA7 | 135112 | chr6:126101898 | IC | 126102306 | 0.165 | 0.0227 |
| NOG | 9241 | chr17:54670370 | ICshore | 54671059 | -0.183 | 0.0227 |
| RRAGA | 10670 | chr9:19049789 | HC | 19049371 | 0.113 | 0.0227 |
| KBTBD4 | 4722 | chr11:47600628 | HC | 47600566 | -0.12 | 0.0227 |
| ZNF623 | 9831 | chr8:144731579 | LC | 144731953 | -0.147 | 0.0227 |
| GABRB3 | 2558 | chr15:27159108 | LC | 27184685 | -0.164 | 0.0227 |
| WHSC1 | 7468 | chr4:1893257 | IC | 1894508 | -0.151 | 0.023 |
| TMEM180 | 79847 | chr10:104220950 | HC | 104221169 | -0.152 | 0.023 |
| OR2V2 | 285659 | chr5:180581900 | LC | 180581942 | -0.153 | 0.0242 |
| LACTB | 114294 | chr15:63414018 | HC | 63413998 | -0.165 | 0.0242 |
| UHRF2 | 115426 | chr9_HCshore:6411902_6414489;  chr9_ICshore:6411820_6414388 | HC | 6413150 | -0.11 | 0.0242 |
| CTU1 | 90353 | chr19_HCshore:51606951_51608203;  chr19_ICshore:51607029_51608162 | HC | 51611646 | -0.114 | 0.0242 |
| NUP43 | 348995 | chr6_HCshore:150067237_150067910;  chr6_ICshore:150067214_150067911 | HC | 150067687 | -0.0884 | 0.0242 |
| CDH10 | 1008 | chr5:24644893 | NA | 24644911 | -0.192 | 0.0245 |
| CYCS | 54205 | chr7:25164393 | HC | 25164979 | 0.183 | 0.0253 |
| C19orf80 | 55908 | chr19:11348389 | IC | 11348125 | -0.184 | 0.0254 |
| C10orf99 | 387695 | chr10:85933748 | LC | 85933553 | -0.146 | 0.0254 |
| DNAH5 | 1767 | chr5:13945990 | LC | 13944588 | -0.176 | 0.0254 |
| HIST1H2AI | 8329 | chr6:27776308 | NA | 27775977 | -0.183 | 0.0255 |
| GALE | 2582 | chr1:24127833 | NA | 24127294 | 0.146 | 0.0255 |
| STAU1 | 6780 | chr20:47804099 | NA | 47804904 | 0.146 | 0.0255 |
| IFT81 | 28981 | chr12:110562699 | HC | 110562139 | -0.123 | 0.0255 |
| BPIFB4 | 149954 | chr20:31670562 | LC | 31669317 | -0.146 | 0.0255 |
| OTUD7A | 161725 | chr15:31947546 | NA | 31947542 | -0.17 | 0.0255 |
| EFHD2 | 79180 | chr1_HCshore:15735794_15737366;  chr1_ICshore:15735682_15737762 | HC | 15736390 | -0.106 | 0.0255 |
| LONP1 | 257062 | chr19_HCshore:5719315_5721498;  chr19_ICshore:5719244_5721592 | HC | 5720462 | -0.118 | 0.026 |
| ZBTB49 | 55646 | chr4:4293029 | LC | 4291923 | -0.269 | 0.0271 |
| PMS2 | 5395 | chr7:6048614 | HC | 6048736 | -0.149 | 0.0275 |
| TMC1 | 117531 | chr9:75136514 | IC | 75136716 | -0.198 | 0.0291 |
| ABHD4 | 63874 | chr14:23067285 | HC | 23067146 | -0.123 | 0.0291 |
| A2ML1 | 144568 | .;chr12_IC:8975165_8975374 | IC | 8975149 | -0.106 | 0.0291 |
| OXA1L | 5018 | chr14:23234922 | IC | 23235730 | -0.169 | 0.0298 |
| PCNP | 57092 | chr3:101293431 | ICshore | 101293041 | -0.312 | 0.0298 |
| ARPC1A | 10552 | chr7_HCshore:98922983_98924236;  chr7_ICshore:98923063_98924233 | HC | 98923495 | -0.12 | 0.0298 |
| RBM47 | 54502 | chr4:40517888 | LC | 40517989 | -0.166 | 0.0299 |
| MEIS1 | 4211 | chr2:66662163 | HC | 66662531 | -0.145 | 0.03 |
| LRRC37BP1 | 147172 | chr17:28934075 | LC | 28935447 | -0.132 | 0.03 |
| OR2K2 | 26248 | chr9:114090211 | IC | 114090712 | -0.154 | 0.0307 |
| HNRNPH3 | 3189 | chr10_HCshore:70090938_70092964;  chr10_ICshore:70090165_70092901 | HC | 70091767 | -0.107 | 0.0307 |
| TCEB2 | 6923 | chr16:2828302 | ICshore | 2827296 | -0.123 | 0.031 |
| SERAC1 | 84947 | chr6:158589306 | HC | 158589311 | 0.194 | 0.0315 |
| POLR3D | 661 | chr8:22102669 | NA | 22102619 | 0.121 | 0.033 |
| NCKAP1L | 3071 | chr12:54891584 | LC | 54891494 | -0.143 | 0.0334 |
| MSMB | 4477 | chr10:51549260 | LC | 51549552 | -0.158 | 0.0338 |
| TRAF3 | 7187 | chr14_HCshore:103242844_103245060;  chr14_ICshore:103242850_103244878 | HC | 103243815 | -0.11 | 0.0338 |
| FAM13C | 220965 | chr10_HCshore:61122014_61122572;  chr10_ICshore:61121881_61122727 | HC | 61122351 | -0.107 | 0.0338 |
| DEF8 | 54849 | .;chr16_ICshore:90013538_90016268 | ICshore | 90015138 | -0.25 | 0.0338 |
| FOLR2 | 2350 | chr11:71927888 | LC | 71927818 | -0.153 | 0.0339 |
| RPUSD2 | 27079 | chr15:40861532 | HC | 40861536 | -0.145 | 0.0339 |
| RCC1 | 1104 | chr1:28832988 | HC | 28832454 | -0.13 | 0.034 |
| SPTAN1 | 6709 | chr9_HCshore:131314002_131315785;  chr9_ICshore:131314107_131315647 | HC | 131314836 | -0.12 | 0.034 |
| FETUB | 26998 | chr3:186356650 | LC | 186358148 | -0.122 | 0.0341 |
| CYP17A1 | 1586 | chr10:104596890 | LC | 104597289 | -0.168 | 0.0342 |
| MRPL54 | 116541 | chr19:3762923 | HC | 3762664 | -0.133 | 0.0342 |
| NGF | 4803 | chr1:115880646 | HC | 115880856 | -0.152 | 0.0346 |
| C9orf24 | 84688 | chr9:34397651 | LC | 34397848 | -0.148 | 0.0351 |
| ROPN1 | 54763 | chr3:123710175 | NA | 123710199 | -0.14 | 0.0354 |
| EFCC1 | 79825 | chr3:128748026 | IC | 128720471 | -0.154 | 0.0354 |
| HLX | 3142 | chr1_HCshore:221051856_221053916;  chr1_ICshore:221051677_221053977 | HC | 221052742 | 0.1 | 0.0354 |
| GNAI1 | 2770 | chr7_HCshore:79763641_79765316;  chr7_ICshore:79763625_79765459 | HC | 79764139 | 0.0967 | 0.0354 |
| DDX18 | 8886 | chr2_HCshore:118571921_118572990;  chr2_ICshore:118571974_118572843 | HC | 118572254 | -0.103 | 0.037 |
| FXYD4 | 53828 | chr10:43867105 | LC | 43867091 | -0.152 | 0.0371 |
| GABRB3 | 2562 | chr15:27019356 | LC | 27018934 | -0.163 | 0.0383 |
| GLRX2 | 51022 | chr1:193075590 | IC | 193075243 | 0.137 | 0.0395 |
| FAM135B | 51059 | chr8:139164635 | IC | 139165458 | -0.148 | 0.0402 |
| SEMA4D | 349236 | chr9:91979776 | LC | 91979565 | -0.177 | 0.0402 |
| RBCK1 | 10616 | chr20:388351 | HC | 388708 | 0.171 | 0.0402 |
| CST1 | 1469 | chr20:23732166 | NA | 23731574 | -0.113 | 0.0402 |
| CDC123 | 8872 | .;chr10_IC:12236699_12237220 | IC | 12237960 | -0.131 | 0.0402 |
| IKBKB | 3551 | chr8_HCshore:42128412_42129300;  chr8_ICshore:42128371_42129504 | HC | 42128819 | -0.101 | 0.0402 |
| RAB3GAP2 | 25782 | chr1_HCshore:220445689_220446258;  chr1_ICshore:220444983_220446120 | HC | 220445842 | -0.0939 | 0.0402 |
| TP53BP2 | 7159 | chr1_HCshore:224032772_224034584;  chr1_ICshore:224032508_224034373 | HC | 224033673 | -0.151 | 0.0402 |
| SURF2 | 6834 | chr9:136224666 | IC | 136223420 | -0.149 | 0.0404 |
| DQ570150 | 25812 | chr22:22986350 | IC | 22985767 | -0.186 | 0.0404 |
| WDR41 | 55255 | chr5_HCshore:76787453_76788783;  chr5_ICshore:76787573_76788633 | HC | 76788331 | -0.0971 | 0.0404 |
| EXOG | 9941 | chr3_HCshore:38537343_38538379;  chr3_ICshore:38537183_38538402 | HC | 38537762 | -0.122 | 0.0411 |
| FAM154A | 158297 | .;chr9_IC:19032509_19033364 | IC | 19033255 | -0.143 | 0.0415 |
| AGRP | 181 | chr16:67517429 | LC | 67517715 | -0.135 | 0.0416 |
| TMTC4 | 84899 | chr13:101295638 | IC | 101294564 | -0.186 | 0.0418 |
| RAD54B | 25788 | chr8:95487224 | NA | 95487310 | 0.134 | 0.0418 |
| ZC2HC1A | 51101 | chr8_HCshore:79577853_79578706;  chr8_ICshore:79577829_79579045 | HC | 79578281 | -0.117 | 0.0418 |
| APBB3 | 10307 | chr5_HCshore:139943675_139944727;  chr5_ICshore:139943588_139944836 | HC | 139944188 | -0.0801 | 0.0418 |
| UVRAG | 7405 | chr11:75526239 | HC | 75526211 | 0.131 | 0.0418 |
| SLC7A11 | 23657 | chr4:139162758 | IC | 139163502 | -0.177 | 0.0418 |
| IFIH1 | 64135 | chr2:163174846 | HC | 163175038 | -0.156 | 0.0418 |
| LRRC7 | 57554 | chr1:70225863 | LC | 70225857 | 0.111 | 0.0418 |
| SPSB1 | 80176 | chr1:9353265 | HC | 9352940 | -0.135 | 0.0418 |
| ZDHHC11 | 79844 | chr5:851372 | IC | 851100 | -0.178 | 0.0418 |
| ZDHHC4 | 55146 | chr7:6617294 | HC | 6617064 | 0.128 | 0.0418 |
| POU3F1 | 5453 | chr1:38511557 | NA | 38512450 | 0.13 | 0.0418 |
| PPP2R5E | 5529 | chr14_HCshore:64009230_64010945;  chr14_ICshore:64008842_64010867 | HC | 64010078 | 0.0968 | 0.0418 |
| LOC642852 | 257103 | chr21:46716835 | LC | 46707966 | -0.177 | 0.0423 |
| RAPSN | 5913 | chr11:47470674 | LC | 47470729 | -0.134 | 0.0423 |
| SCRN2 | 90507 | .;chr17_IC:45917575_45919377 | IC | 45918698 | -0.0868 | 0.0423 |
| CCDC24 | 149473 | chr1:44457109 | HC | 44457279 | -0.152 | 0.0427 |
| TCIRG1 | 10312 | chr11:67806668 | IC | 67806461 | -0.104 | 0.0432 |
| PTPDC1 | 138639 | chr9:95832383 | HC | 96793075 | 0.156 | 0.0435 |
| NR0B2 | 8431 | .;chr1_IC:27240168_27240377 | IC | 27240566 | -0.148 | 0.0435 |
| TRAF5 | 7188 | chr1:211499588 | IC | 211499956 | -0.135 | 0.0439 |
| SF1 | 7536 | chr11_HCshore:64544821_64546797;  chr11_ICshore:64544940_64546776 | HC | 64546315 | -0.109 | 0.0439 |
| AK097836 | 56255 | chr20:8000564 | HC | 8000548 | 0.131 | 0.044 |
| LIN37 | 55957 | .;chr19_IC:36239041_36240240 | IC | 36239261 | -0.0769 | 0.044 |
| ZC3HAV1 | 56829 | chr7_HCshore:138793613_138794932;  chr7_ICshore:138792827_138795016 | HC | 138794464 | -0.0988 | 0.044 |
| C3orf35 | 339883 | chr3:37427383 | LC | 37427799 | 0.12 | 0.0442 |
| GSTA5 | 221357 | chr6:52711444 | LC | 52710892 | -0.179 | 0.0445 |
| POLN | 353497 | chr4:2231610 | LC | 2230969 | -0.195 | 0.0463 |
| SULT2B1 | 6820 | chr19:49055431 | LC | 49055428 | -0.221 | 0.0467 |
| KIAA1737 | 85457 | chr14_HCshore:77563707_77565064;  chr14_ICshore:77563882_77565146 | HC | 77564577 | -0.0889 | 0.0467 |
| C5 | 727 | chr9:123811564 | LC | 123812553 | -0.152 | 0.0474 |
| ATP5E | 514 | chr20_HCshore:57606692_57608176;  chr20_ICshore:57606826_57608145 | HC | 57607421 | -0.16 | 0.0474 |
| BRD7 | 29117 | chr16:50402689 | NA | 50402829 | -0.0996 | 0.0481 |
| SLC5A12 | 159963 | chr11:26744417 | LC | 26744973 | -0.125 | 0.0482 |
| SYS1 | 55861 | chr20:43991296 | ICshore | 43991739 | 0.229 | 0.0483 |
| NQO1 | 1728 | chr16:69760708 | LC | 69760532 | -0.163 | 0.0483 |
| MFAP1 | 4236 | chr15:44117132 | ICshore | 44116950 | 0.12 | 0.0483 |
| IER3 | 8870 | chr6:30712467 | HC | 30712326 | 0.124 | 0.0483 |
| PLTP | 5360 | chr20:44540621 | NA | 44540786 | 0.134 | 0.0483 |
| HAVCR2 | 84868 | chr5:156536057 | LC | 156536247 | 0.107 | 0.0493 |
| RELA | 5970 | chr11:65429415 | IC | 65430442 | -0.181 | 0.0493 |
| CD47 | 961 | chr3_HCshore:107809282_107810925;  chr3_ICshore:107809091_107810911 | HC | 107809934 | -0.136 | 0.0494 |

*^1^*CpG density surrounding each interrogated CpG site/island. HC, high-density CpG island; IC, intermediate-density CpG island; ICshore, IC that overlaps with some HC; LC, non-island.

*^2^*Value of the coefficient of the linear model associated with (MUFA+PUFA)/SFA

*^3^*P-value calculated by moderated t-statistics and adjusted for multiple comparisons according to Benjamini and Hochberg.
