# Supplementary material for: Dietary fat quality impacts genome-wide DNA methylation patterns in a cross-sectional study of Greek preadolescents
Source: Eur J Hum Genet. 2014 Jul 30;23(5):654–62. doi: 10.1038/ejhg.2014.139 (PMC4402618; doi:10.1038/ejhg.2014.139)
Supplement: Supplementary Table 4 [file ejhg2014139x4.doc]

**Supplementary table 4. Information on the significant pathways associated with PUFA/SFA, and (MUFA+PUFA)/SFA. The list of the genes associated with the significant CpG sites/islands associated with PUFA/SFA or with (MUFA+PUFA)/SFA was input in the CPDP over-representation analysis, with all genes from the Illumina Infinium HumanMethylation27 Bead-Chip array as background. We selected the pathways with a p-value<0.05 and a q-value<0.05.**

| Pathway name | Candidate genes contained*^1^* | P-value*^2^* | Q-value*^2^* | Database |
| --- | --- | --- | --- | --- |
| ***PUFA/SFA*** | | | | |
| Leptin | FYN, PDE3A, NCOA1 | 0.000176 | 0.00584 | NetPath |
| TGF_beta_Receptor | NCOA1, SKI, BRCA1, SMAD6 | 0.000249 | 0.00617 | NetPath |
| Signaling by BMP | SKI, SMAD6 | 0.000555 | 0.00728 | Reactome |
| BMP signalling pathway | SKI, SMAD6 | 0.000620 | 0.00728 | HumanCyc |
| Doxorubicin Pathway (Cardiomyocyte Cell), Pharmacodynamics | CYCS, CBR1 | 0.000834 | 0.00784 | PharmGKB |
| BMP receptor signaling | SKI, SMAD6 | 0.00224 | 0.0155 | PID |
| TGF-Ncore | SKI, SMAD6 | 0.00290 | 0.0155 | Signalink |
| Integrated Pancreatic Cancer Pathway | BRCA1, CCNA2, SMAD6 | 0.00368 | 0.0155 | Wikipathways |
| EGF-Ncore | FYN, SMAD6 | 0.00412 | 0.0155 | Signalink |
| Mechanism of gene regulation by peroxisome proliferators via ppara | NCOA1, NR2F1 | 0.00428 | 0.0155 | BioCarta |
| Mechanism of gene regulation by peroxisome proliferators via ppara | NCOA1, NR2F1 | 0.00428 | 0.0155 | PID |
| Viral myocarditis - Homo sapiens (human) | FYN, CYCS | 0.00462 | 0.0155 | KEGG |
| TGF Beta Signaling Pathway | SKI, SMAD6 | 0.00462 | 0.0155 | Wikipathways |
| Leptin signaling pathway | NCOA1, FYN | 0.00535 | 0.0158 | Wikipathways |
| ATF-2 transcription factor network | CCNA2, BRCA1 | 0.00573 | 0.0158 | PID |
| Validated nuclear estrogen receptor alpha network | BRCA1, NCOA1 | 0.00573 | 0.0158 | PID |
| DNA Damage Response | CYCS, BRCA1 | 0.00693 | 0.0170 | Wikipathways |
| E2F transcription factor network | CCNA2, BRCA1 | 0.00758 | 0.0170 | PID |
| IL6 | FYN, NCOA1 | 0.00758 | 0.0170 | NetPath |
| miRNA Regulation of DNA Damage Response | CYCS, BRCA1 | 0.00758 | 0.0170 | Wikipathways |
| Regulation of nuclear SMAD2/3 signaling | SKI, NCOA1 | 0.00825 | 0.0176 | PID |
| Progesterone-mediated oocyte maturation (human) | PDE3A, CCNA2 | 0.0102 | 0.0208 | KEGG |
| Androgen receptor signaling pathway | NCOA1, BRCA1 | 0.0112 | 0.0219 | Wikipathways |
| Purine nucleotides nucleosides metabolism | ERGR, PDE3A | 0.0139 | 0.0262 | INOH |
| Cholinergic synapse - Homo sapiens (human) | CHRNA6, FYN | 0.0173 | 0.0312 | KEGG |
| TGF beta super family signaling pathway canonical | SKI, SMAD6 | 0.0179 | 0.0312 | INOH |
| Developmental Biology | FYN, SCN2A, NCOA1 | 0.0193 | 0.0323 | Reactome |
| Generic Transcription Pathway | ZNF212, SKI, NR2F1 | 0.0200 | 0.0323 | Reactome |
| BCR | FYN, CYCS | 0.0227 | 0.0356 | NetPath |
| Regulation of toll-like receptor signaling pathway | RBCK1, SMAD6 | 0.0237 | 0.0360 | Wikipathways |
| Adipogenesis | NR2F1, NCOA1 | 0.0260 | 0.0380 | Wikipathways |
| Hepatitis B - Homo sapiens (human) | CCNA2, CYCS | 0.0275 | 0.0380 | KEGG |
| AndrogenReceptor | NCOA1, BRCA1 | 0.0275 | 0.0380 | NetPath |
| Purine metabolism - Homo sapiens (human) | PRPS1L1, PDE3A | 0.0319 | 0.0428 | KEGG |
| ***(MUFA+PUFA)/SFA*** | | | | |
| TWEAK | TRAF3, TRAF5, RELA, IKBKB | 0.000287 | 0.0370 | NetPath |
| NF-κB is activated and signals survival | NGF, RELA, IKBKB | 0.000313 | 0.0370 | PID |
| NF-κB is activated and signals survival | NGF, RELA, IKBKB | 0.000313 | 0.0370 | Reactome |
| human TAK1 activates NFkB by phosphorylation and activation of IKKs complex | RELA, IKBKB | 0.000401 | 0.0370 | PID |
| p75NTR signals via NF-kB | NGF, RELA, IKBKB | 0.000632 | 0.0466 | Reactome |

*^1^*Name of the genes from the input gene list that are involved in the corresponding pathway.

*^2^*P-value and q-value calculated according to the hypergeometric test
