# Supplementary figures and images for: Dietary fat quality impacts genome-wide DNA methylation patterns in a cross-sectional study of Greek preadolescents
Source: Eur J Hum Genet. 2014 Jul 30;23(5):654–62. doi: 10.1038/ejhg.2014.139 (PMC4402618; doi:10.1038/ejhg.2014.139)

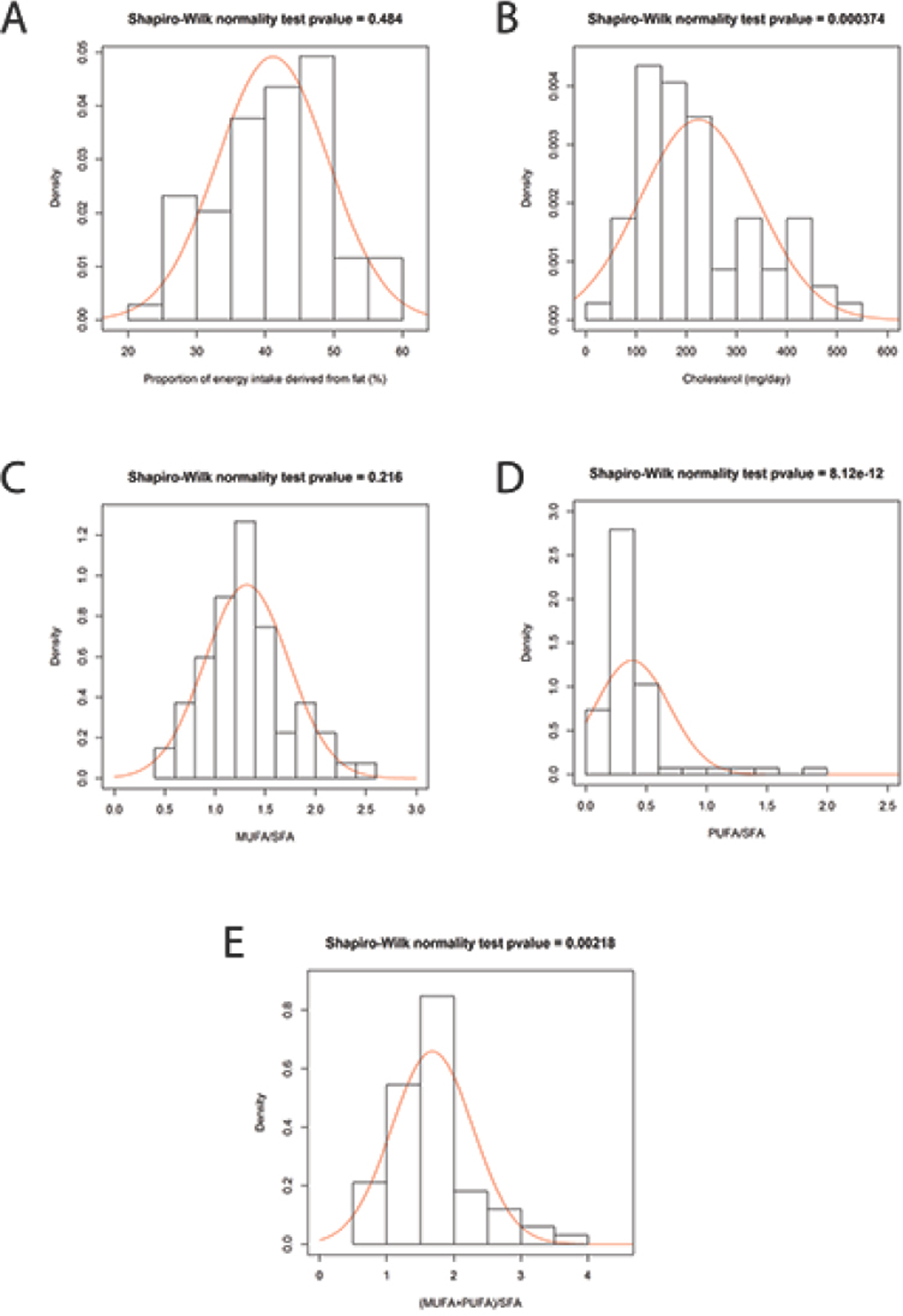

Supplement: Supplementary Figure 1 [file ejhg2014139x5.tif]
