# Supplementary material for: Dietary fat quality impacts genome-wide DNA methylation patterns in a cross-sectional study of Greek preadolescents
Source: Eur J Hum Genet. 2014 Jul 30;23(5):654–62. doi: 10.1038/ejhg.2014.139 (PMC4402618; doi:10.1038/ejhg.2014.139)
Supplement: Supplementary Figure 1 Legend [file ejhg2014139x6.doc]

**Additional figure 1. Distributions of proportion of energy intake derived from fat (A), cholesterol (B), MUFA/SFA (C), PUFA/SFA (D) and (MUFA+PUFA)/SFA (E).**
